# Naringenin Inhibits Platelet Activation and Arterial Thrombosis Through Inhibition of Phosphoinositide 3-Kinase and Cyclic Nucleotide Signaling

**DOI:** 10.3389/fphar.2021.722257

**Published:** 2021-08-12

**Authors:** Manting Huang, Minzhen Deng, Wenqiang Nie, Dezhi Zou, Huanlin Wu, Danping Xu

**Affiliations:** ^1^ Department of Vascular Intervention, Zhongshan Hospital of Traditional Chinese Medicine, Affiliated to Guangzhou University of Chinese Medicine, Zhongshan, China; ^2^ Guangdong Provincial Hospital of Chinese Medicine, The Second Clinical College of Guangzhou University of Chinese Medicine, Guangzhou, China; ^3^ Department of Traditional Chinese Medicine, The First Affiliated Hospital of Guangdong Pharmaceutical University, Guangzhou, China; ^4^ Emergency Department, The First Affiliated Hospital of Sun Yat-sen University, Guangzhou, China; ^5^ Dongzhimen Hospital, Beijing University of Chinese Medicine, Beijing, China; ^6^ Department of Traditional Chinese Medicine, The Eighth Affiliated Hospital, Sun Yat-sen University, Shenzhen, China

**Keywords:** platelet, arterial thrombosis, PI3K/AKT, cGMP, naringenin

## Abstract

Citrus flavanoids intake can reduce the risk of cardiovascular diseases. Naringenin, a natural predominant flavonoid abundant in citrus fruits, possesses protective effects against atherothrombotic diseases. As platelet activation plays central roles in atherothrombogenesis, we studied the effects of naringenin on platelet activation, signaling, thrombosis and hemostasis. Naringenin dose-dependently inhibited agonist-induced platelet aggregation *in vitro*, and exhibited more-potent efficacy on ADP-induced platelet aggregation. It also suppressed platelet aggregation stimulated by ADP *ex vivo*. Naringenin inhibited ADP-induced platelet α-granule secretion, fibrinogen binding, intracellular calcium mobilization and platelet adhesion on collagen-coated surface. Naringenin also inhibited platelet spreading on fibrinogen and clot retraction, processes mediated by outside-in integrin signaling. Mechanism studies indicated that naringenin suppressed PI3K-mediated signaling and phosphodiesterase activity in platelets, in addition to increasing cGMP levels and VASP phosphorylation at Ser239. Furthermore, naringenin-induced VASP phosphorylation and inhibition of platelet aggregation were reversed by a PKA inhibitor treatment. Interestingly, naringenin inhibited thrombus formation in the (FeCl_3_)-induced rat carotid arterial thrombus model, but not cause a prolonged bleeding time in mice. This study suggests that naringenin may represent a potential antiplatelet agent targeting PI3K and cyclic nucleotide signaling, with a low bleeding risk.

## Introduction

Platelets play central roles in both physiological hemostasis and pathological thrombosis. Inappropriate activation of platelets is initiated upon some pathological conditions, such as the rupture of an unstable atherosclerotic plaque, which triggers thrombotic occlusions of coronary or cerebral arteries, and thus resulting in myocardial infarction and stroke. Accordingly, antiplatelet therapy has been established as a cornerstone to treat atherothrombotic diseases. Currently approved antiplatelet drugs are hampered by associated side effects including increased bleeding risk, and therefore much priority has been given to the development of safer and more efficacious antithrombotic drugs. The relationship between citrus flavanoids intake and a decreased incidence of cardiovascular diseases has been well established by a number of epidemiological studies. Citrus flavanoids show anti-inflammatory, insulin-sensitizing, lipid-lowering and antihypertensive properties in cell culture and animal models, as well as several clinical studies. Several citrus flavonoids, such as tangeretin and nobiletin, show marked suppression of platelet activation through the impairment of key platelet signaling (Vaiyapuri et al., 2013; Vaiyapuri et al., 2015).

Naringenin, a natural predominant flavonoid abundant in the peel of citrus fruits, has powerful and diverse biological and pharmacological properties (Zeng et al., 2018). Accumulating studies have suggested that naringenin is capable of inhibiting inflammatory response in various cell types and animal models, and therefore may be developed as an immunomodulator in the treatment of inflammation-related disorders (Zeng et al., 2018). Naringenin has been reported to improve the renal failure and platelet purinergic signaling alterations in high-cholesterol fed rats by inhibiting ROS and NF-κB signaling pathways (Chtourou et al., 2016). A recent study has shown that naringenin supplementation to a chow diet increases energy expenditure and fatty acid oxidation, and prevents adiposity in lean, pair-fed Ldlr^−/-^ mice (Burke et al., 2019). Naringenin has also been demonstrated to inhibit collagen-stimulated platelet aggregation and thrombus formation (Wright et al., 2013), but the detailed mechanisms have not been investigated. In the present study, we demonstrate the antiplatelet and antithrombotic activities of naringenin without bleeding diathesis, which inhibits platelet activation and thrombus formation by regulation of platelet signaling, blockade of phosphoinositide 3-kinase (PI3K)/protein kinase B (Akt) pathway, along with elevation of cyclic guanosine monophosphate (cGMP) generation leading to vasodilator-stimulated phosphoprotein (VASP) phosphorylation.

## Materials and Methods

### Animals

Male Sprague-Dawley (SD) rats (7 weeks age, 240–250 g) and male Kunming (KM) mice (7 weeks age) were obtained from the Experimental Animal Center of Guangdong Province, and were housed in climate-controlled quarters (22–26°C at 40–70% humidity) with a 12 h light/dark cycle and free access to food and water. All animal experiments were approved by the Animal Care and Use Committee of Guangzhou University of Chinese medicine (Permit number: 2016001). All animal care and procedures conform to the ‘Guide for the Care and Use of Laboratory Animal’ as promulgated by the National Institute of Health.

### Chemicals and Reagents

Naringenin and naringin were purchased from the Division of Chinese Material Medica and Natural Products, National Institute for the Control of Pharmaceutical and Biological Products (NICPBP, Beijing, China). Adenosine diphosphate (ADP), Thrombin, apyrase, fibrinogen, Fluo 3-AM, TRITC-Phalloidin, BSA, Triton X-100, HEPES, LY294002, H89, Rp-8-Bromo-β-phenyl-1, N2-ethenoguanosine 3´, 5´-cyclic mono-phosphorothioate sodium salt (*R*p-8-Br-PET-cGMPS) and GF109203X were all purchased from Sigma (St. Louis, MO, United States). Collagen was purchased from Chrono-Log Corp (Havertown, PA, United States). CD62P (P-selectin) antibody was obtained from BD Biosciences (San. Jose, CA, United States). FITC-conjugated anti-fibrinogen antibody, cyclic adenosine monophosphate (cAMP) and cGMP ELISA kits were purchased from Cayman chemical (Ann Arbor, MI). Antibodies against GAPDH, Akt, phospho-Akt (Ser473), phospho-Akt (Thr308), p44/42 MAPK (ERK1/2), phospho-p44/42 MAPK (ERK1/2) (Thr^202^/Tyr^204^), glycogen synthase kinase 3β (GSK3β), phospho- GSK3β (Ser9), VASP, phospho-VASP (Ser239), phosphor-VASP (Ser157) were acquired from Cell Signaling Technology (Beverly, MA, United States). The PI3-Kinase p110 beta antibody was from Abcam (Cambridge, United Kingdom). All chemicals were of reagent grade.

### *In Vitro* Platelet Aggregation

Washed rat platelets were prepared as described previously (Gao et al., 2011) with a minor modification. Briefly, rat blood was drawn from the abdominal aorta into the siliconized vacutainers containing 1:9 (v/v) 3.8% sodium citrate, supplemented with 100 ng/ml prostaglandin E1 (PGE1) and centrifuged at 300 × *g* for 6 min at room temperature. Platelet-rich plasma (PRP) was obtained, and after the addition of 100 ng/ml PGE1, platelets were pelleted at 900 × *g* for 10 min. Platelets were washed once in Tyrode’s buffer (12 mM NaHCO_3_, 138 mM NaCl, 5.5 mM glucose, 2.9 mM KCl, 2 mM MgCl_2_, 0.42 mM NaH_2_PO_4_, 10 mM HEPES, pH 7.4) containing 100 ng/ml PGE1, and finally resuspended in Tyrode’s buffer containing 0.02 U/mL apyrase to a final concentration of 4×10^8^ cells/mL. Washed platelets were preincubated with vehicle (0.4% dimethyl sulfoxide) or various concentrations of naringenin in the presence of 2 mM CaCl_2_ for 5 min at 37°C, and then further stimulated by ADP (10 μM), thrombin (0.2 U/mL) or collagen (2 μg/ml). The maximum platelet aggregation rate was detected within 5 min with continuous stirring.

### *Ex Vivo* Platelet Aggregation

Male SD rats were randomly divided into 5 groups with 6 rats in each group. The rats were orally administered naringenin (200, 400, and 800 mg/kg), clopidogrel (7.875 mg/kg) or vehicle (0.5% CMC-Na) once a day for 7 days. Sixty minutes after the last sample treatment, blood was drawn from the abdominal aorta of rats into the siliconized vacutainers containing 1:9 (v/v) 3.8% sodium citrate. The collected blood was centrifuged at 100 × *g* for 10 min at room temperature to obtain PRP. Then, platelet-poor plasma (PPP) was obtained by following centrifugation of the remainder at 1,600 × *g* for 10 min. The platelet counts of PRP were adjusted to 4×10^8^ cells/mL using PPP. PRP was incubated for 5 min at 37°C and then stimulated by ADP (5 μM). The maximum platelet aggregation rate was determined as mentioned above.

### Measurement of Naringenin Cytotoxicity

To assess the potential cytotoxicity of naringenin, lactate dehydrogenase (LDH) leakage from platelets were determined. Washed platelets were preincubated with either various concentrations of naringenin or vehicle at 37°C for 5 min and then centrifuged at 350 × *g* for 3 min at room temperature. The supernatant was detected by LDH assay kit (Beyotime Biotechnology, Shanghai, China). LDH leakage was expressed as percentages of the total LDH leakage in platelets completely lysed with 0.1% Triton X-100.

### Flow Cytometry

Flow cytometric assays were performed as described previously (Wang et al., 2019). ADP-stimulated P-selectin exposure and fibrinogen binding were determined in washed platelets. Washed platelets were preincubated with different concentrations of naringenin or vehicle in the presence of 2 mM CaCl_2_ for 5 min at 37°C, and then stimulated with ADP (10 μM) for 5 min. The platelets were immediately incubated with PE-labeled CD62P or FITC-labeled fibrinogen in the dark at room temperature for 15 min. The cells were finally fixed with 1% (v/v) paraformaldehyde prior to analysis using an Accuri C6 flow cytometer (BD Biosciences, United States).

Calcium mobilization in ADP-induced platelets was measured using flow cytometry. Washed platelets were incubated with Fluo 3-AM (5 μM) at 37°C for 30 min and washed. The Fluo 3-loaded platelets were preincubated with vehicle or different concentrations of naringenin at 37°C for 5 min in the presence of 1 mM CaCl_2_ and then stimulated with ADP (10 μM) for 5 min. The fluorescence intensity was analyzed using an Accuri C6 flow cytometer.

### Platelet Spreading on Fibrinogen

Washed platelets were incubated with vehicle or various concentrations of naringenin at 37°C for 10 min in the presence of 2 mM CaCl_2_ and allowed to spread on fibrinogen-coated coverslips for 45 min at 37°C (Liu et al., 2016). After washed three times with phosphate-buffered saline (PBS), adherent platelets were fixed with 4% paraformaldehyde, permeabilized with 0.1% Triton X-100 and stained with TRITC-conjugated phalloidin (1 μg/ml) in the dark at room temperature for 60 min. Adherent platelets were observed under an inverted fluorescence microscope (Nikon TE-2000S) and processed using Image J software.

### Clot Retraction

PRP was incubated with vehicle or different concentrations of naringenin for 10 min at 37°C. Clot retraction was initiated by adding fibrinogen (400 μg/ml) and thrombin (1 U/mL), and allowed to proceed at 37°C. Images were taken 15 min later.

### Platelet Adhesion on Collagen-Coated Surface

Washed platelets were treated with naringenin or vehicle at 37°C for 10 min in the presence of 2 mM CaCl_2_ (Suzuki-Inoue et al., 2001). The platelets were added to collagen-coated coverslips and incubated at 37°C for 45 min. After unbound platelets were removed by washing, adherent platelets were fixed with 4% paraformaldehyde, permeabilized with 0.1% Triton X-100 and stained with TRITC-conjugated phalloidin (1 μg/ml). Adherent platelets were viewed using an inverted fluorescence microscope (Nikon TE-2000S) and processed using Image J software.

### Immunoblotting

For *in vitro* platelet aggregation, aliquots of washed platelets were preincubated with vehicle or various concentrations of naringenin for 5 min and stimulated with ADP (10 μmol/L) under stirring at 37°C for 5 min. For *ex vivo* platelet aggregation, PRP was incubated at 37°C for 5 min and stimulated with ADP (5 μmol/L) under stirring at 37 °C. The platelet aggregation was terminated by adding ethylenediaminetetraacetic acid (EDTA; 10 mM). Total protein was prepared using RIPA lysis buffer and protein content was determined by bicinchoninic acid (BCA) protein assay kit. Equal amounts of platelet proteins (30 μg) were separated on 10% SDS polyacrylamide gel electrophoresis (SDS-PAGE) and then transferred to poly vinylidene difluoride (PVDF) membranes and subjected to western blot. Blots were blocked with TBS-Tween 20 containing 5% BSA and probed with primary antibodies diluted in blocking solution at 4°C overnight followed by incubation with the corresponding HRP secondary antibodies. The protein bands were visualized using enhanced chemiluminescence (ECL).

### Measurements of cAMP and cGMP

Washed platelets were preincubated with vehicle or various concentrations of naringenin in the presence of 2 mM CaCl_2_ at 37°C for 5 min and then stimulated with ADP (10 μM) for 5 min for platelet aggregation. The reaction was terminated by adding EDTA (10 mM). The platelets were boiled for 5 min at 100 °C followed by centrifugation at 13,000 × *g* for 5 min cAMP and cGMP levels in the supernatant were measured using cAMP or cGMP enzyme immunoassay kits (Cayman Chemicals, Ann Arbor, MI, United States).

### Assay for Phosphodiesterase Activity in Platelets

The effects of naringenin on PDE activity were determined using high-performance liquid chromatography (HPLC) (Hu et al., 2011). Briefly, PRP was pelleted by centrifugation for 10 min at 900 × *g* and homogenized in PBS containing 0.1% Triton X-100 using an ultrasonic homogenizer. After centrifugation at 12,000 × *g* for 20 min at 4°C, the supernatant was subjected to PDE activity assay.

DMSO vehicle or different concentrations naringenin were added to assay buffer (137 mM NaCl, 2.7 mM KCl, 8.8 mM Na_2_HPO_4_, 1.5 mM KH_2_PO_4_, 1 mM CaCl_2_, 1 mM MgCl_2_, pH 7,4) containing cAMP or cGMP. The reaction was initiated by adding 30 μL PDE extracts prepared from rat PRP as described above. Some extracts were boiled for 3 min to inactivate PDE before addition. After incubation for 30 min at °C, the reaction was terminated by boiling the mixture for 3 min. Subsequently, the mixture was cooled on ice followed by centrifugation at 12,000 × *g* for 20 min at 4°C. The supernatant was subjected cAMP or cGMP assay using HPLC. PDE activity was expressed as percentages of the hydrolysis of cAMP or cGMP.

### FeCl_3_-Induced Carotid Arterial Thrombus Model in Rats

To determine the effects of naringenin on thrombogenesis, a FeCl_3_-induced carotid arterial thrombus model was performed as reported previously with some modifications (Eckly et al., 2011). Briefly, naringenin (200, 400, and 800 mg/kg), clopidogrel (7.875 mg/kg) or vehicle (0.5% CMC-Na) was administered orally to rats once a day for 7 days. The rats were anaesthetized using 1% pentobarbital sodium salt and fixed supinely after the last administration. The left carotid artery was exposed and thrombosis was induced by applying a Whatman filter paper (5 mm × 1 mm) saturated with 10% (w/v) FeCl_3_ on the top of the left carotid artery for 5 min. After removal of the filter paper, the artery was washed with PBS and the blood flow was continuously monitored for 20 min with a miniature Doppler flowprobe (1 mm diameter) using a Transonic Model TS420 flowmeter (Transonic Systems, Ithaca, NY, United States). Time to occlusion of the carotid artery was defined as a blood flow of 0 ml/min for 3 min. When the blood flow does not cease within 20 min, the occlusion time is recorded as 20 min. At the end of the experiment, the left carotid artery was dissected. A haematoxylin and eosin stain was performed on the artery.

### Tail Bleeding Time in Mice

Mice were orally administered naringenin (280, 560, and 1,120 mg/kg), clopidogrel (11.375 mg/kg) or vehicle (0.5% CMC-Na) once a day for 7 days. Sixty minutes after the last administration, mice were anaesthetized with 1% pentobarbital sodium salt and placed on a 37°C heating pad. The tail was transected at a site 3 mm proximal to the tip and immediately immersed into saline kept at 37°C. The time to bleeding cessation (defined as no bleeding for 30 s) was measured. When the bleeding time lasted longer than 10 min, the bleeding was stopped, and the bleeding time was recorded as 10 min.

### Plasma Coagulation Assay in Rats

Rats received intragastric administration of naringenin (200, 400, and 800 mg/kg), clopidogrel (7.875 mg/kg) or vehicle (0.5% CMC-Na), once a day for 7 days. Sixty minutes after the last treatment, the rats were anaesthetized with 1% pentobarbital sodium salt and blood was drawn from the abdominal aorta into the siliconized vacutainers containing 1:9 (v/v) 3.8% sodium citrate. After the PPP was prepared as described above, the prothrombin time (PT), activated partial thromboplastin time (APTT), thrombin time (TT) and fibrinogen (FIB) were measured using a coagulometer.

### Dosage Information

In our *in vitro* studies, concentrations of naringenin (100, 200, 400, and 800 μM) used were based on our previously published study (Huang et al., 2017), and consistent with or below those given in other studies (Chattopadhyay et al., 2016; Wang et al., 2018). These doses had no cytotoxic effects on platelets as measured by LDH leakage from platelets (Table 1). Rats in our studies were received naringenin (200, 400, or 800 mg/kg/day) suspended in 0.5% CMC-Na *via* oral gavage between 8:00 a.m. and 10:00 a.m. for 7 days. The doses were based on previously published studies (Arul and Subramanian, 2013; Lin et al., 2014).

**TABLE 1:** Effects of naringenin on cytotoxicity of platelets.

Naringenin (μM)	0	100	200	400	800	1,600
Cytotoxicity (%)	0.92 ± 0.54	0.99 ± 0.21	1.16 ± 0.39	2.11 ± 0.81	2.50 ± 0.85	3.47 ± 1.41

Data are expressed as means ± SD (*n* = 6). No significant changes were observed.

### Statistical Analysis

All data are expressed as means ± SD. Individual measurements of naringenin effects were analysed by one-way ANOVA followed by either LSD or Newman-Keuls *post hoc* test using SPSS 17.0 statistical package (SPSS, Inc., Chicago, IL). The vessel occlusion time and tail bleeding time were analysed using the nonparametric Kruskal-Wallis global test using GraphPad Prism 5.0 from GraphPad Software Inc. *p* < 0.05 considered to be statistically significant.

## Results

### Naringenin Inhibits Agonist-Induced Platelet Aggregation

**FIGURE 1:**
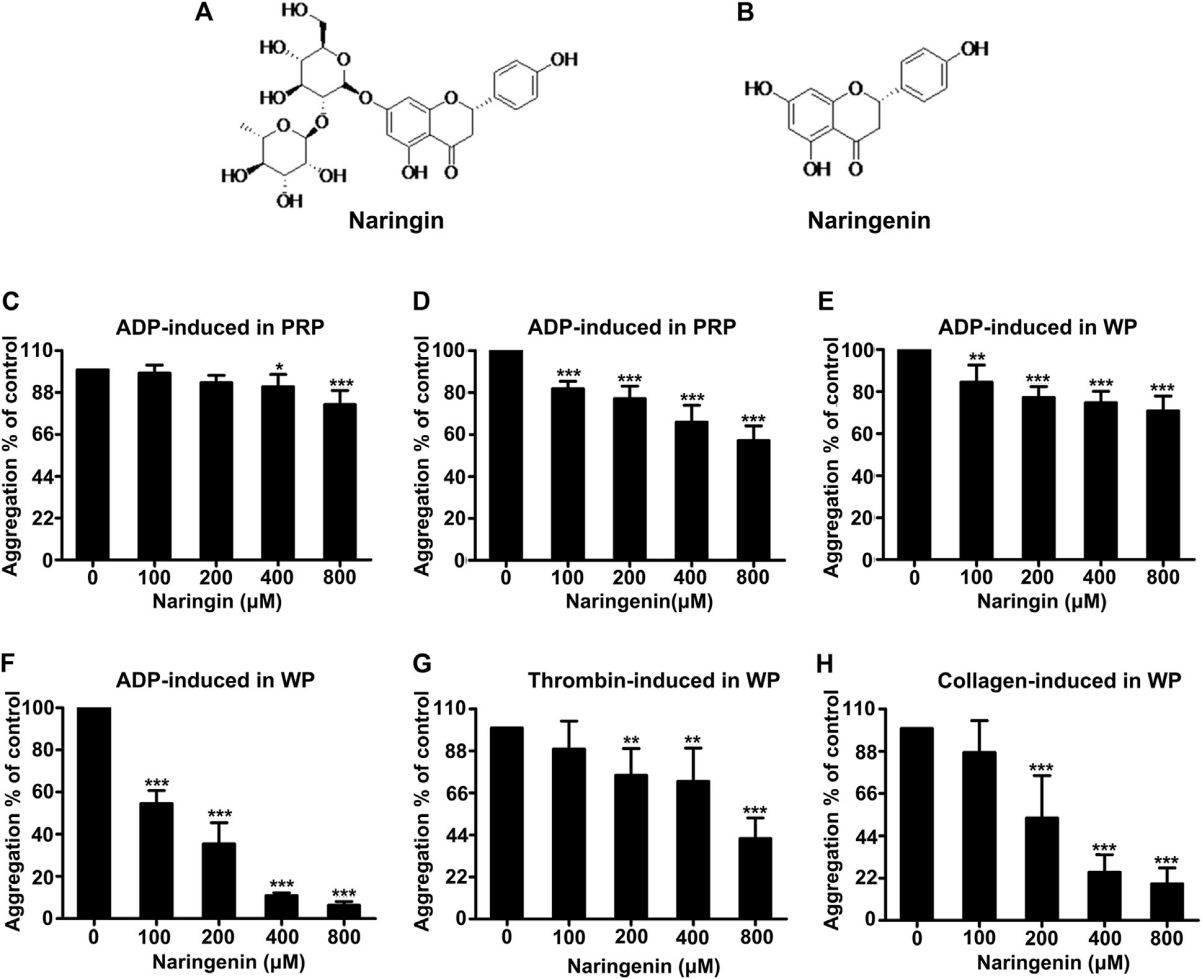
Effects of naringenin and naringin on platelet aggregation. Chemical structure of naringin **(A)** and naringenin **(B)**. The effects of naringin **(C)** or naringenin **(D)** on PRP were measured following stimulation with 10 μmol/L ADP. Rat washed platelet aggregation performed with or without different concentrations of naringin **(E)** or naringenin **(F)** was recorded after stimulation with 10 μmol/L ADP. Similarly, Washed platelet aggregation performed with vehicle or various concentrations of naringenin was recorded after stimulation with 0.2 U/mL thrombin **(G)** or 2 μg/ml collagen **(H)**. WP represents washed platelets. Data are expressed as means ± SD (*n* = 6), the level of aggregation obtained at 5 min with vehicle-treated samples (0 μM) was taken as 100%. *p < 0.05, **p < 0.01, ***p < 0.001 compared with the vehicle control group.

Platelet aggregation was induced by ADP using washed platelets and PRP. Washed platelets were preincubated with vehicle (containing 0.4% DMSO) or different doses (100, 200, 400, and 800 μM) of naringenin or naringin for 5 min before activation with ADP (10 μM) for 5 min. Both naringenin and naringin revealed a dose-dependent inhibition in platelet aggregation (Figures 1E,F). The more effective inhibitor of ADP-induced platelet aggregation was naringenin. For example, naringenin, at doses of 100 and 800 μM, decreased ADP-induced platelet aggregation to 54.5 ± 6.2% and 6.3 ± 1.7%, respectively (*n* = 6; *p* < 0.001, control group platelet aggregation was taken as 100%) (Figure 1F). Naringenin was able to cause significant levels of inhibition in ADP (5 μM)- stimulated platelet aggregation, and naringenin (200, 400 and 800 μM) exerted similar antiplatelet efficacy (Supplementary Figure S1). However, 800 μM naringin only reduced platelet aggregation to 70.8 ± 7.1% in ADP-induced aggregation of washed platelets (Figure 1E). At 800 μM naringenin exerted a 11-fold greater level of inhibition (*p* < 0.001) than naringin, indicating that the A ring C-7 hydroxyl may be essential for potent platelet inhibition (Figures 1A,B). Because dietary flavonoids have been reported to bind plasma proteins and cause the lowering of inhibitory potency in PRP (Wright et al., 2013), the ability of naringenin and naringin on platelet function in PRP was investigated to compare their effects with washed platelets. Similarly, both naringenin (100–800 μM) and naringin (400–800 μM) significantly inhibited ADP-induced aggregation of PRP, but the levels of inhibition were decreased (Figures 1C,D). For example, 100 μM naringenin reduced the aggregation of washed platelets by 45%, while in PRP this was decreased by 18%. Moreover, naringenin (200–800 μM) significantly inhibited thrombin- and collagen-induced aggregation of washed platelets (Figures 1G,H), with IC_50_ values of 684.3 ± 80.8 μM and 236.2 ± 33.1 μM, respectively, while naringenin inhibited ADP-induced washed platelets aggregation with an IC_50_ value of 118.5 ± 10.4 μM. These results indicated that naringenin was more efficient at inhibiting ADP-induced platelet aggregation than other agonist-induced platelet aggregations.

### Naringenin Inhibits ADP-Induced Platelet α-Granule Secretion and Integrin αIIbβ3-Mediated Inside-out Signaling

**FIGURE 2:**
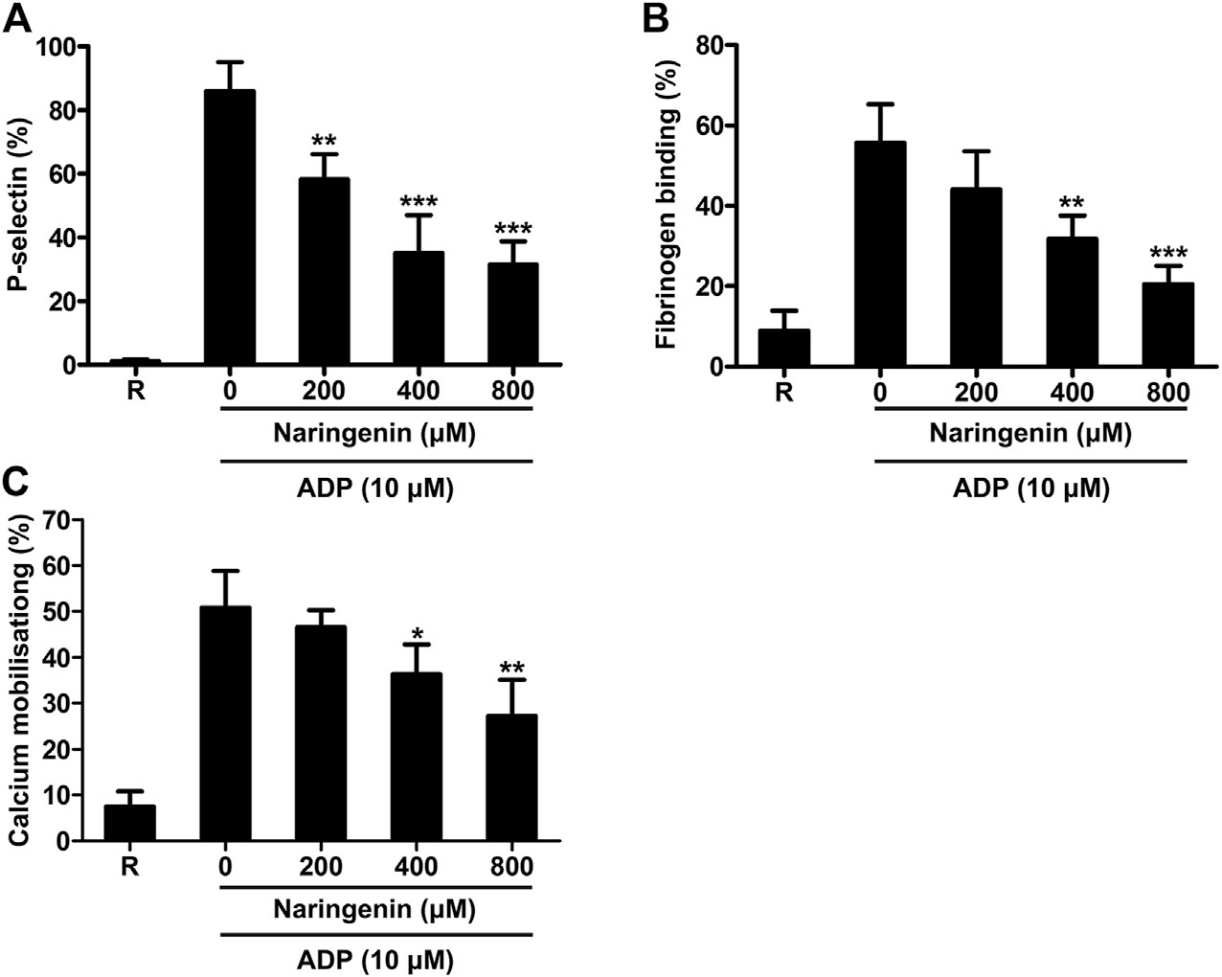
Naringenin inhibits platelet granule secretion, integrin inside-out signaling and calcium mobilization. The effects of naringenin on P-selectin exposure **(A)** and fibrinogen binding **(B)** upon stimulation with ADP (10 μmol/L) were determined in washed platelets. Fluo-3 AM dye-labeled washed platelets were stimulated with ADP (10 μmol/L) in the absence or presence of different concentrations of naringenin, and the intracellular calcium levels were detected by flow cytometry **(C)**. R represents the levels of P-selectin, fibrinogen binding or calcium mobilization in resting platelets. Data are expressed as means ± SD (*n* = 3). *p < 0.05, **p < 0.01, ***p < 0.001 compared with the vehicle control group.

Platelet activation leads to degranulation, which further boosts platelet activation signals through the paracrine/autocrine actions and is considered to be an important step towards thrombus formation (Mackman, 2008). Platelet α-granules contain P-selectin, vWF, fibrinogen and so on. The activation of platelets leads to the transfer of P-selectin in α-granules to the membrane surface of platelets. P-selectin exposure on the platelet surface is an important indicator of platelet-activation cascade. Therefore, the effect of naringenin on ADP-activated platelet granule secretion was determined by measuring P-selectin exposure (a marker for α-granule secretion) in washed platelets using flow cytometry. Naringenin significantly inhibited ADP-induced P-selectin exposure (Figure 2A). Naringenin at doses of 200, 400, and 800 μM reduced P-selectin exposure from 86.06 ± 9.05% in the absence of naringenin to 58.33 ± 7.86, 35.12 ± 11.91, and 31.56 ± 7.26%, respectively (*p* < 0.01, *p* < 0.001 and *p* < 0.001, respectively).

The binding of agonists to platelet receptors triggers the conformational change of integrin αIIbβ3 through inside-out signaling, leading to facilitating plasma fibrinogen binding and consequent platelet aggregation (Huang et al., 2019). To investigate the effects of naringenin on intetrin inside-out signaling induced by ADP, we performed fibrinogen binding (a marker for αIIbβ3 inside-out signaling) assays using flow cytometry in the presence or absence of naringenin. As shown in Figure 2B, ADP (10 μM) increased fibrinogen binding in washed platelets from 8.90 ± 4.97% at baseline to 55.67 ± 9.62%. Consistent with platelet aggregation, the increases were significantly reduced to 31.74 ± 5.85% and 20.48 ± 4.63% (*n* = 3; *p* < 0.01 and *p* < 0.001, respectively) in the presence of 400 and 800 μM naringenin. These results suggest that naringenin inhibits αIIbβ3 inside-out signaling that are required for platelet aggregation.

### Naringenin Inhibits Calcium Mobilization in ADP-Induced Platelet.

Elevation of intracellular calcium levels in platelets play an important role in platelet activation, including shape change, degranulation, integrin αIIbβ3 affinity modulation, aggregation and thrombus formation (Flaumenhaft, 2016). Therefore, the effects of naringenin on intracellular calcium mobilization were measured in washed platelets using flow cytometry. As shown in Figure 2C, naringenin (400 and 800 μM) significantly prevented the mobilization of intracellular calcium induced by ADP, suggesting that naringenin modulates cytoplasmic calcium levels to inhibit platelet activation.

### Naringenin Inhibits Integrin αIIbβ3-Mediated Outside-in Signaling

**FIGURE 3:**
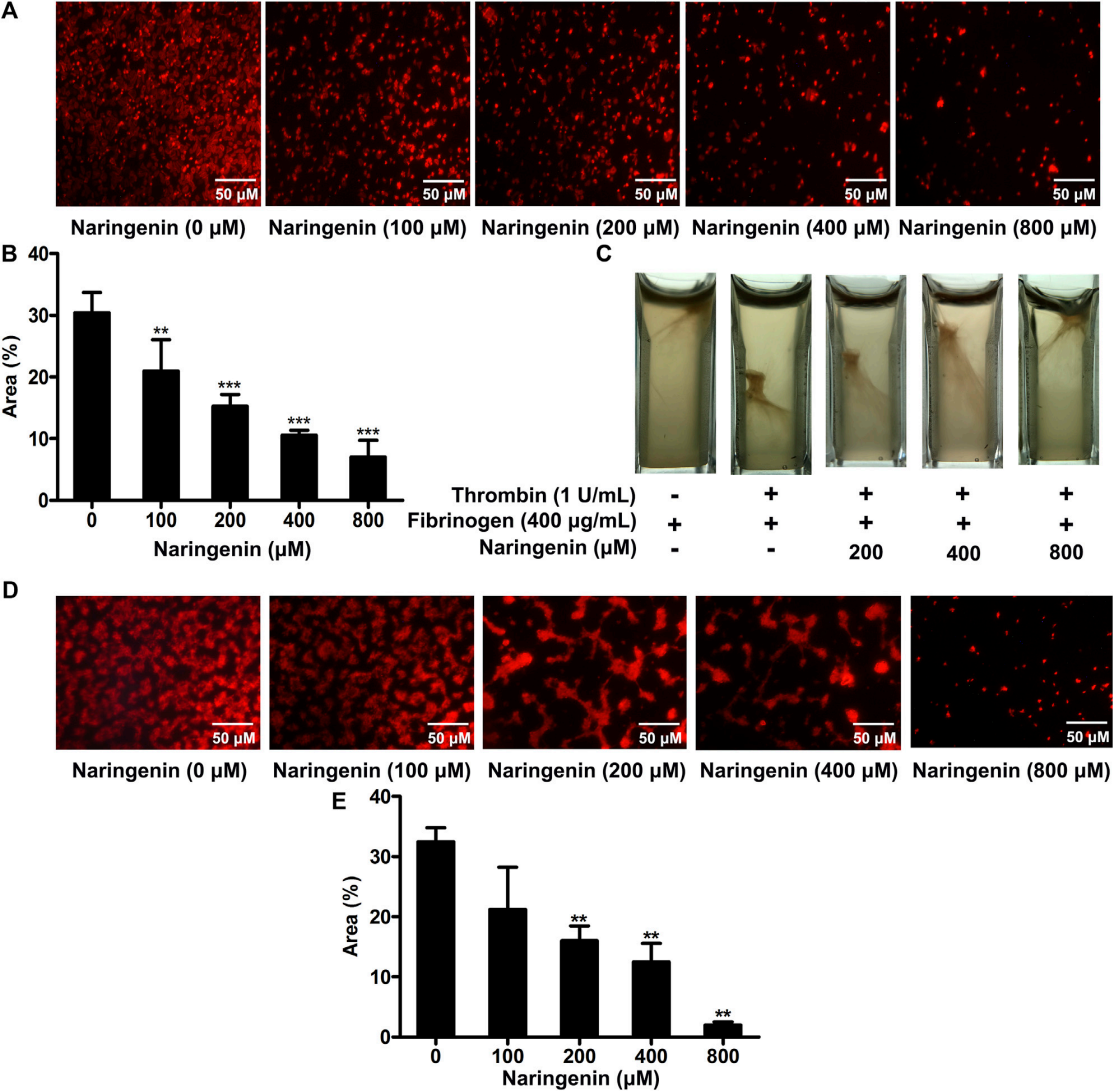
Naringenin inhibits integrin outside-in signaling and platelet adhesion on collagen-coated surfaces. Washed platelets were preincubated with various concentrations of naringenin or vehicle for 10 min at 37°C, then allowed to spread on fibrinogen-coated surface. **(A)** Representative images from one of three independent experiments with similar results. The percentages of the average surface area of individual platelets were plotted **(B)**. PRP was incubated with naringenin (200, 400 and 800 μmol/L) or vehicle for 10 min at 37°C. Fibrinogen (400 μg/ml) was added and fibrin clot formation was initiated by the addition of thrombin (1 U/mL). Photographs of the clots were taken 15 min later. Similar results were obtained in three independent experiments **(C)**. Washed platelets were preincubated with various concentrations of naringenin or vehicle for 10 min at 37°C, then allowed to adhere on collagen-coated coverslips. **(D)** Representative images from one of three independent experiments with similar results. The percentages of the average surface area of individual platelets were plotted **(E)**. Data are expressed as means ± SD (*n* = 3). **p < 0.01, ***p < 0.001 compared with the vehicle control group.

As naringenin had inhibitory effects on platelet aggregation and fibrinogen binding, reflecting platelet inside-out signaling, we further investigated whether naringenin regulated integrin αIIbβ3-mediated outside-in signaling through the measurement of platelet spreading on immobilized fibrinogen and clot retraction (Huang et al., 2019). Staining platelets with TRITC-conjugated phalloidin showed that naringenin-treated platelets had less spreading on fibrinogen-coated surface than vehicle-treated platelets (Figure 3A). As shown in Figure 3B, the average surface coverage of vehicle-treated platelets was 30.37 ± 3.31%. Naringenin (100, 200, 400, and 800 μM) markedly inhibited platelet spreading in a dose dependent manner and reduced the surface coverage to 20.96 ± 5.08, 15.29 ± 1.87, 10.56 ± 0.79, and 6.98 ± 2.74%, respectively (*p* < 0.01, *p* < 0.001, *p* < 0.001 and *p* < 0.001, respectively).

Platelet activation triggers clot retraction as a late consequence of integrin outside-in signaling. Platelet clot retraction depends on both extracellular fibrinogen-integrin interaction and integrin-cytoskeletal association (Huang et al., 2016). To further investigate the effects of naringenin on platelet function, we performed platelet clot retraction assay by adding thrombin to PRP in the absence or presence of naringenin (200, 400, and 800 μM), and the subsequent platelet-dependent clot retraction was monitored for 15 min at 37°C. As shown in Figure 3C, clot retraction was significantly reduced in the presence of naringenin-treated platelets compared to the control samples. These results suggest that naringenin impairs αIIbβ3 outside-in signaling which modulates the process of platelet spreading on fibrinogen and clot retraction.

### Naringenin Inhibits Platelet Adhesion on Collagen-Coated Surfaces

Platelet adhesion and subsequent aggregation at the vascular injury site was initiated by the exposed subendothelial collagen and von Willebrand factor (vWF) (Poulte et al., 2017). The modulatory effects of naringenin on platelet adhesion on a collagen-coated surface were analysed using staining platelets with TRITC-conjugated philloidin. Although the platelet adhesion was normal in vehicle treated samples, platelet adhesion was significantly reduced in the presence of naringenin (200, 400 and 800 μM) compared to the control samples (Figures 3D,E). Most naringenin-treated platelets display poor adhesion on collagen-coated surfaces, suggesting that naringenin is able to inhibit initial platelet adhesion.

### Naringenin Inhibits PI3K-Mediated Signaling in ADP-Induced Platelets

**FIGURE 4:**
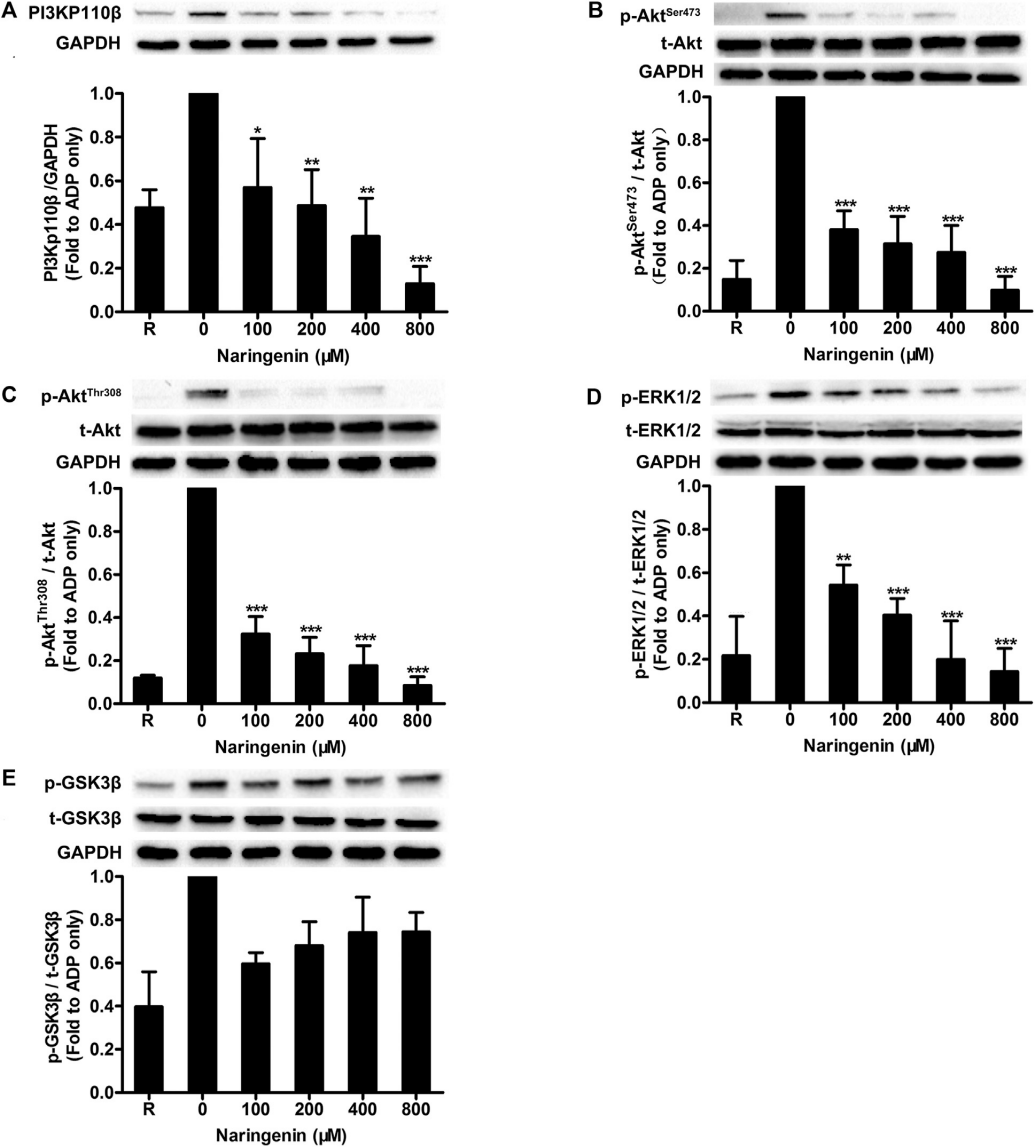
Naringenin inhibits PI3K-mediated signaling in ADP-induced platelets. Washed rat platelets were preincubated without or with naringenin at 37°C for 5 min, and then stimulated with ADP (10 μmol/L) under stirring for 5 min. Platelet proteins were then extracted and analyzed by immunoblotting using PI3Kp110β antibody **(A)** and phospho-specific antibodies such as Akt^Ser473^ **(B)**, Akt^Thr308^ **(C)**, ERK1/2^Thr202/Tyr204^ **(D)** and GSK3β^Ser9^ **(E)**. Total level of GAPDH was measured on each sample as a loading control. The blots shown are representative of three separate experiments. The relative protein expression levels were quantified by ImageJ 1.47v software. The level of relative protein expression obtained with vehicle control (0 μM) was taken as 1. R represents the level of relative protein expression in resting platelets. Data are expressed as means ± SD (*n* = 3). *p < 0.05, **p < 0.01, ***p < 0.001 compared with the vehicle control group.

Because naringenin had potent inhibitory effects on ADP-stimulated platelet aggregation, and PI3K plays an important role in maintaining ADP-mediated platelet aggregation, we investigated the effects of naringenin on PI3K signaling in platelets. The activation of PI3K was detected by measuring the expression of PI3Kp110β, which plays an important role in platelet function during arterial thrombosis and has been developed as a new target for antithrombotic drugs (Jackson et al., 2005). Results showed that naringenin (100–800 μM) significantly inhibited PI3Kp110β expression induced by ADP in a dose-dependent manner (Figure 4A). Akt is downstream of PI3K. Therefore, the activation of PI3K was also determined by measuring phosphorylation of Akt at Ser 473 and Thr 308. Naringenin markedly decreased phosphorylation of Akt at both Ser 473 and Thr 308 sites induced by ADP (Figures 4B,C), indicating that naringenin action on the PI3K/Akt pathway. In addition, ADP-induced extracellular signal-regulated kinase (ERK) phosphorylation was significantly suppressed by naringenin in a dose-dependent manner (Figure 4D). It is known that was important for ADP-induced ERK activation (Garcia et al., 2010). GSK3β is a downstream substrate of Akt. We found that naringenin had no influence on GSK3β phosphorylation (Figure 4E). Therefore, these data indicate that naringenin inhibits platelet activation involving PI3K-mediated platelet signaling.

**FIGURE 5:**
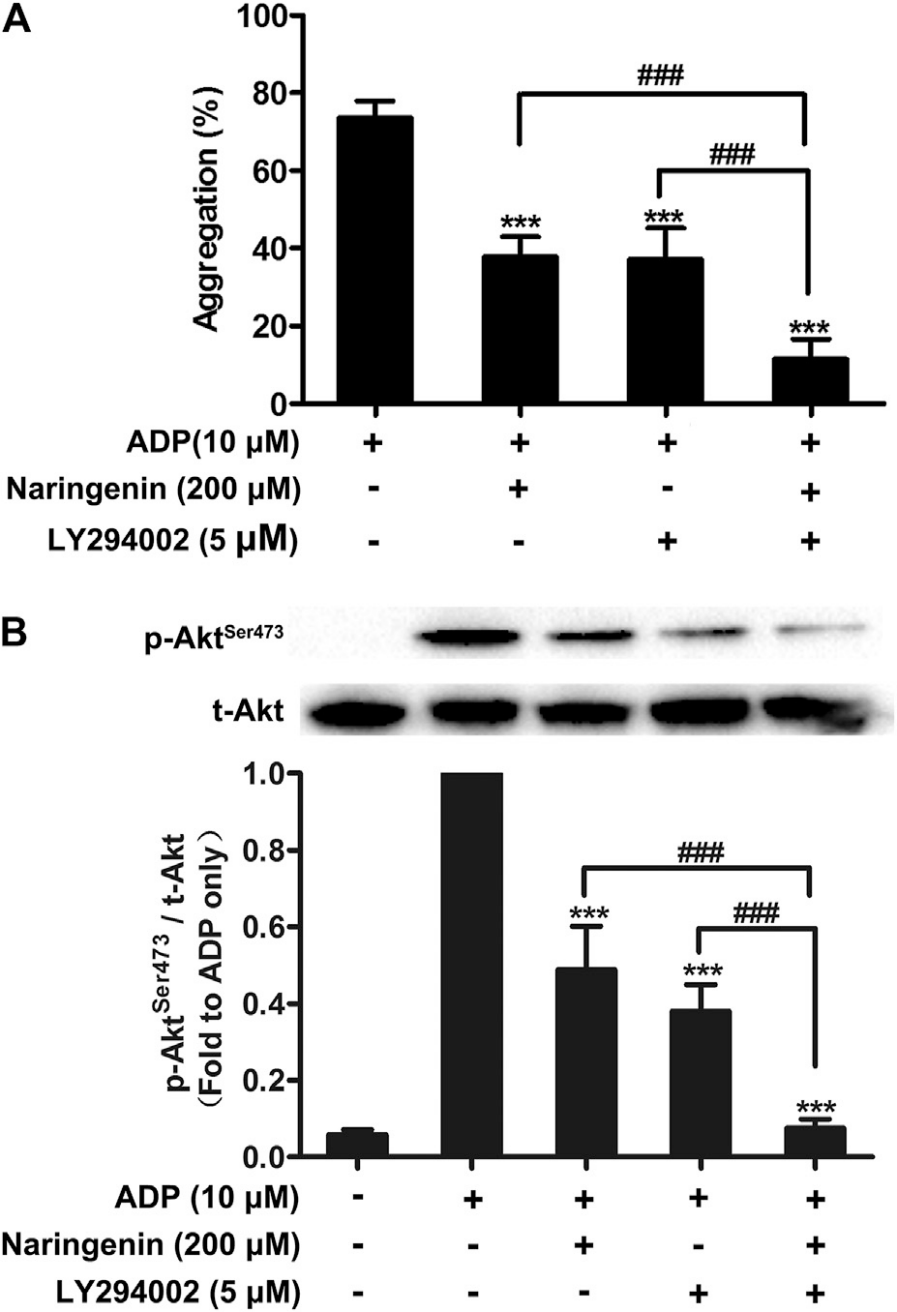
Effects of naringenin combined with PI3K pan inhibitor LY294002 on platelet aggregation and Akt phosphorylation. Washed rat platelets were preincubated with naringenin (200 μmol/L) alone, the PI3K pan inhibitor LY294002 (5 μmol/L) alone or both for 5 min prior to ADP (10 μmol/L) activation. After stimulation with ADP, platelet aggregation was quantified and expressed as a percentage **(A)**. Data are expressed as means ± SD (*n* = 6). ***p < 0.001 compared with the ADP-treated group, ^###^ *p* < 0.001 compared with naringenin alone or LY294002 alone. Platelet proteins were then extracted and specific antibodies were used to measure the level of total and phosphorylated Akt **(B)**. The blots shown are representative of three separate experiments. The relative protein expression levels were quantified by ImageJ 1.47v software. The level of phosphorylation obtained in the ADP-treated group was taken as 1. Data are expressed as means ± SD (*n* = 3). ***p < 0.001 compared with the ADP-treated group, ^###^ *p* < 0.001 compared with naringenin alone or LY294002 alone.

To further investigate how naringenin acts on PI3K pathway, washed platelets were preincubated with low-dose naringenin (200 μM), the pan-PI3K inhibitor LY294002 or both to determine platelet aggregation and Akt phosphorylation. Naringenin and LY294002 were found to inhibit ADP-induced platelet aggregation similarly, and naringenin and LY294002 demonstrated additive inhibition when combined (Figure 5A). In accordance with its inhibition of platelet aggregation, naringenin had an additive inhibitory effect with LY294002 on ADP-induced Akt^Ser 473^ phosphorylation (Figure 5B).

### Naringenin Elevates Platelet cGMP Levels and Inhibits Phosphodiesterase Activity

**FIGURE 6:**
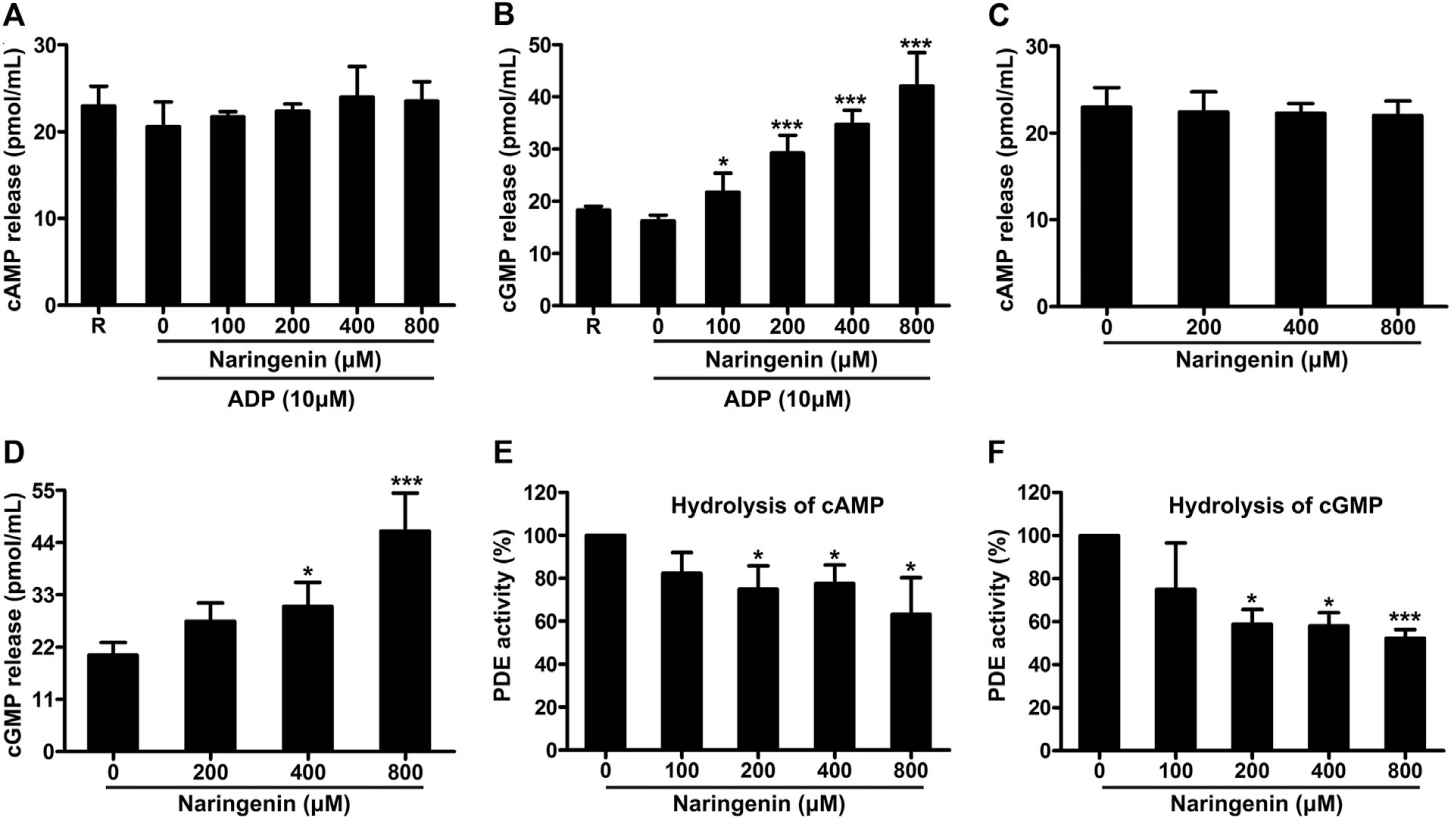
Naringenin elevates platelet cGMP levels and inhibits phosphodiesterase activity. The levels of cAMP **(A)** and cGMP **(B)** in washed platelets preincubated with vehicle or naringenin (100, 200, 400, and 800 μmol/L) were determined after stimulation with ADP (10 μmol/L) using ELISA kits. Similarly, the levels of cAMP **(C)** and cGMP **(D)** were determined in resting platelets incubated with naringenin (200, 400, and 800 μmol/L) or vehicle for 5 min. R represents the levels of cAMP or cGMP in resting platelets. Data are expressed as means ± SD (*n* = 4). *p < 0.05, ***p < 0.001 compared with the vehicle control group. The effects of naringenin on phosphodiesterase activity were measured by HPLC on the hydrolysis of cAMP **(E)** and cGMP **(F)**. The level of phosphodiesterase activity obtained with vehicle control (0 μM) was taken as 100%. Data are expressed as means ± SD (*n* = 3). *p < 0.05, ***p < 0.001 compared with the vehicle control group.

It is known that cyclic nucleotides (cAMP and cGMP) generation and the resulting protein kinases activation prevent platelet activation induced by various stimuli. Therefore, the effects of naringenin on the levels of cAMP and cGMP on ADP stimulation in washed platelets were measured in the absence or presence of naringenin (100, 200, 400, and 800 μM). As shown in Figures 6A,B, naringenin elevated cGMP levels significantly in ADP-activated platelets but not cAMP levels at all doses used, indicating that naringenin-mediated inhibition of platelet activation is dependent on the elevation of cGMP and not on cAMP. To further clarify the elevation of cGMP levels by naringenin depends on ADP activation, the levels of cGMP were determined in resting platelets preincubated with or without naringenin (200, 400, and 800 μM) for 5 min. Similarly, naringenin (400 and 800 μM) increased basal cGMP levels but did not significantly alter cAMP gerneration (Figures 6C,D). These data indicate that naringenin-mediated cGMP elevation is not dependent on agonist stimulation or platelet activation.

The levels of cAMP and cGMP are modulated by PDE, which hydrolyze cAMP and cGMP to terminate cyclic nucleotide signaling. Since naringenin increased cGMP levels, we next determined whether naringenin affects PDE activity by HPLC using cGMP and cAMP as substrates. Naringenin (200–800 μM) was found to significantly inhibit PDE activity on the hydrolysis of either cAMP (Figure 6E) or cGMP (Figure 6F), indicating that naringenin may elevate cGMP levels through decreased hydrolysis of this cyclic nucleotide by PDE.

### Naringenin Promotes VASP Phosphorylation

**FIGURE 7:**
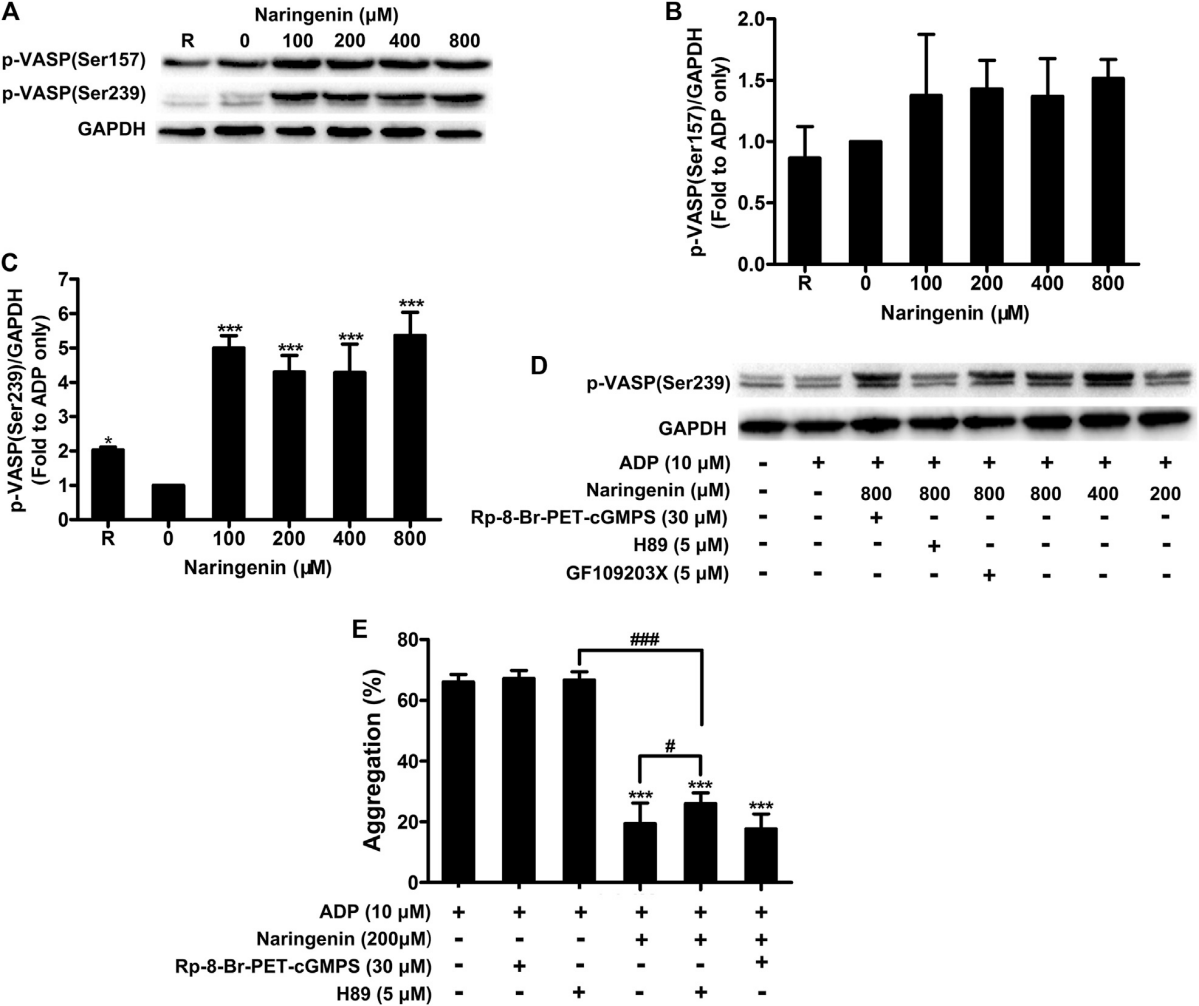
Naringenin promotes VASP phosphorylation. The effects of naringenin on phosphorylation of VASP (at positions Ser 157 and Ser239) were measured in washed platelets upon stimulation with ADP (10 μmol/L) by western blotting **(A)**. The blots shown are representative of three separate experiments. The phosphorylation levels were quantified by ImageJ 1.47v software. The level of phosphorylation obtained with vehicle control (0 μM) was taken as 1 **(B,C)**. R represents the phosphorylation level in resting platelets. Data are expressed as means ± SD (*n* = 3). *p < 0.05, ***p < 0.001 compared with the vehicle control group. VASP phosphorylation at Ser239 was also analyzed in washed platelets incubated with 5 μmol/L PKA (H89), 30 μmol/L PKG (Rp-8-Br-PET-cGMPS), or 5 μmol/L PKC (GF109203X) inhibitors before treatment with naringenin **(D)**. Washed platelets were incubated with vehicle or naringenin (200 μmol/L) at 37°C in the presence or absence of 5 μmol/L PKA (H89) or 30 μmol/L PKG (Rp-8-Br-PET-cGMPS) inhibitors. After treatment, platelets were stimulated with ADP (10 μmol/L) to trigger aggregation **(E)**. Data are expressed as means ± SD (*n* = 6). ***p < 0.001 compared with the ADP-treated group, ^#^ *p* < 0.05, ^###^ *p* < 0.001 compared with naringenin alone or H89 alone.

VASP is a substrate for cAMP- and cGMP- dependent protein kinases, which mediate cAMP- and cGMP-dependent inhibitory signaling in platelets, respectively. Increased cAMP levels followed by the activation of cAMP-dependent protein kinase A (PKA) phosphorylates VASP at position Ser157, whereas the elevation of cGMP leads to VASP phosphorylation at position Ser239 by cGMP-dependent protein kinase G (PKG) (Aszódi et al., 1999; Li et al., 2003a). To further examine whether naringenin modulates cAMP- or cGMP-mediated inhibitory signaling, the phosphorylation of VASP (Ser157 and Ser239) in washed platelets on stimulation with ADP were measured in the absence or presence of naringenin (100, 200, 400, and 800 μM). Consistent with its elevation of cGMP levels, naringenin induced the phosphorylation of VASP at Ser239 in ADP-stimulated platelets (Figures 7A,C), but did not significantly alter the phosphorylation of position Ser157 (Figures 7A,B). Incubation of platelets with 5 μmol/L PKA inhibitor (H89) before treatment with naringenin inhibited the increase in phosphorylation of VASP at Ser239 (Figure 7D). However, only small changes were observed in phosphorylation of VASP at Ser239 in platelets incubated with either PKG inhibitor (*R*p-8-Br-PET-cGMPS; 30 μmol/L) or protein kinase C (PKC) inhibitor (GF109203X; 5 μmol/L) before treatment with naringenin (Figure 7D), indicating that naringenin-mediated phosphorylation of VASP at Ser293 is dependent on the elevation of cGMP- and PKA-dependent signaling. To further confirm the antiplatelet effects of naringenin through PKA-dependent signaling, the effects of naringenin on platelet aggregation were measured in the presence of PKG inhibitor (*R*p-8-Br-PET-cGMPS) and PKA inhibitor (H89). As shown in Figure 7E, PKA inhibitor (H89; 5 μmol/L) did not affect the level of platelet aggregation, but its pre-treatment significantly restored platelet aggregation levels when incubated with naringenin in ADP-stimulated platelets. Moreover, the preincubation of PKG inhibitor (*R*p-8-Br-PET-cGMPS; 30 μmol/L) did not affect the final extent of platelet aggregation when incubated with naringenin. These results imply that the antiplatelet effects of naringenin may be achieved through elevated cGMP levels and PKA-dependent signaling.

### Effects of Naringenin on Platelet Aggregation and Intracellular Signaling *Ex Vivo*

**FIGURE 8:**
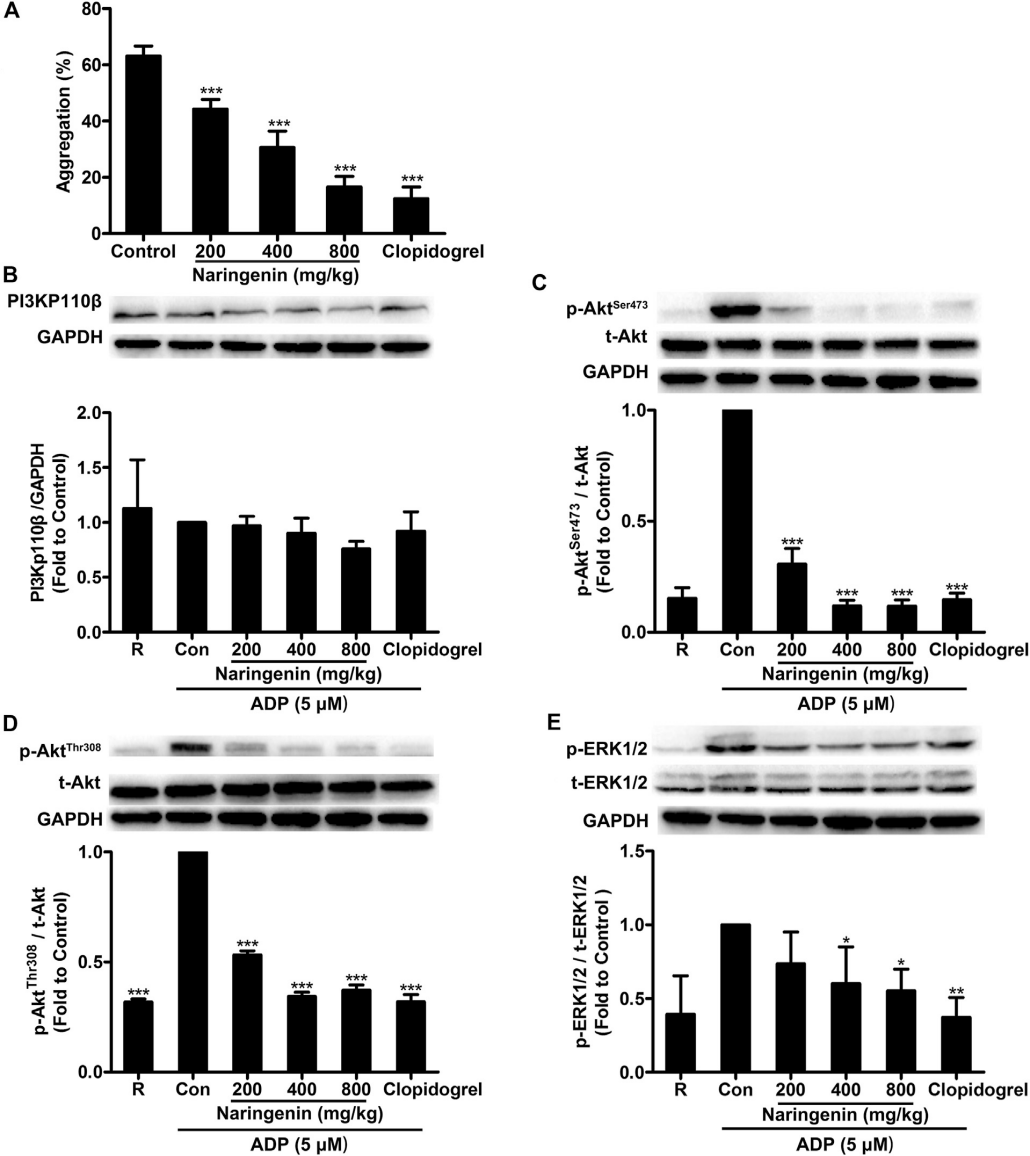
Intragastric administration of naringenin inhibits ADP-induced rat platelet aggregation and platelet PI3K signaling phosphorylation. Rats were orally administered naringenin (200, 400, and 800 mg/kg), clopidogrel (7.875 mg/kg) or vehicle (0.5% CMC-Na) for 7 days. Blood was collected and PRP was obtained after the last treatment. Platelet aggregation was induced by ADP (5 μmol/L) **(A)**. Data are expressed as means ± SD (*n* = 6). After ADP stimulation, platelet proteins were then extracted and analyzed by immunoblotting using PI3Kp110β antibody **(B)** and phospho-specific antibodies such as Akt^Ser473^ **(C)**, Akt^Thr308^ **(D)** and ERK1/2^Thr202/Tyr204^ **(E)**. Total level of GAPDH was measured on each sample as a loading control. The blots shown are representative of three separate experiments. The relative protein expression levels were quantified by ImageJ 1.47v software. The level of relative protein expression obtained with vehicle control was taken as 1. R represents the level of relative protein expression in resting platelets. Data are expressed as means ± SD (*n* = 3). *p < 0.05, **p < 0.01, ***p < 0.001 compared with the vehicle control group (Con).

We have demonstrated that naringenin showed potent inhibitory effects on platelet aggregation *in vitro*. To further confirm the antiplatelet effects of naringenin, an *ex vivo* test on rats was performed. Consistent with its inhibition of platelet aggregation *in vitro*, naringenin potently inhibited ADP-induced platelet aggregation in a concentration-dependent manner *ex vivo*. Oral administration of naringenin (200, 400, and 800 mg/kg) to rats for 7 days significantly inhibited ADP-induced platelet aggregation from 63.2 ± 3.6 to 44.3 ± 3.4, 30.6 ± 5.9, and 16.6 ± 3.8% (Figure 8A), respectively. At a dose of 800 mg/kg, naringenin exerted similar antiplatelet efficacy as that of clopidogrel at 7.875 mg/kg.

To further investigate the antiplatelet mechanisms of naringenin *ex vivo*, several signaling molecules such as PI3Kp110β, Akt and ERK were detected. After oral administration of naringenin (200, 400, and 800 mg/kg) to rats for 7 days, rat PRP was prepared and stimulated by ADP (5 μmol/L), and platelets were lysed to determine protein phosphorylation levels using western blot. In accordance with naringenin-mediated inhibition of PI3K signaling in ADP-induced platelets *in vitro*, intragastric administration of naringenin significantly suppressed the phosphorylation of Akt (at both Ser473 and Thr308 sites) and ERK in rat PRP on stimulation with ADP (Figures 8C–E), but only modest changes in expression of PI3Kp110β (Figure 8B). In addition, clopidogrel at 7.875 mg/kg also showed potency for the phosphorylation of Akt (at Ser473 and Thr308) and ERK that was similar to 800 mg/kg narigenin. These data indicate that naringenin inhibits ADP-induced platelet aggregation *ex vivo* by blocking the PI3K-mediated platelet signaling.

### Effects of Naringenin on Arterial Thrombosis in Rats

**FIGURE 9:**
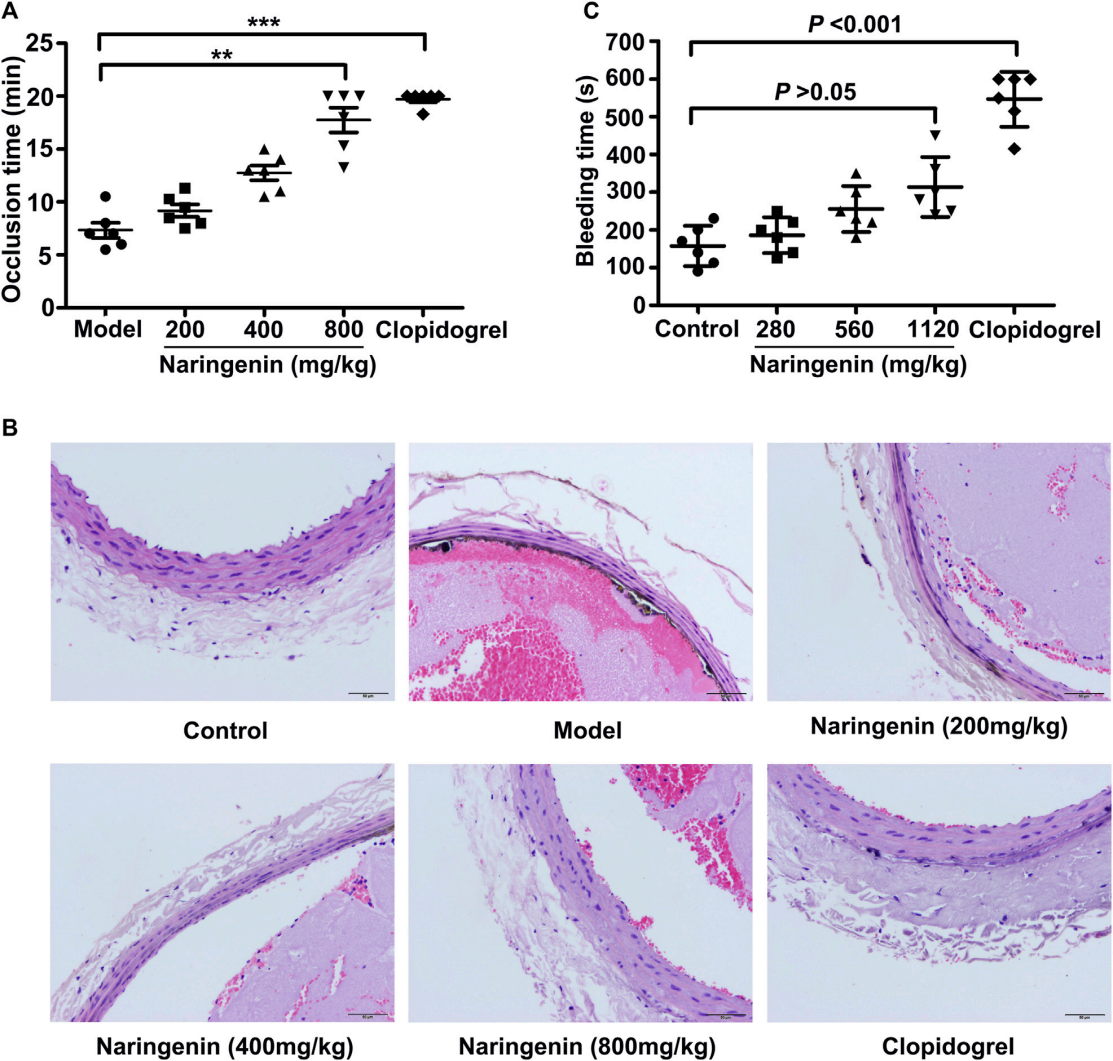
Naringenin inhibits arterial thrombosis formation without bleeding risk. Arterial thrombosis was induced by 10% FeCl_3_ on the common carotid artery of rats that were orally treated with naringenin (200, 400, and 800 mg/kg), clopidogrel (7.875 mg/kg) or vehicle (0.5% CMC-Na) for 7 days. Time to occlusion of the carotid artery after FeCl_3_ injury for 5 min was measured using a Transonic Model TS420 flowmeter **(A)**. Representative sections of H&E-stained carotid artery from rats treated as labeled **(B)**. Magnification: 10×20. Naringenin (280, 560, and 1,120 mg/kg), clopidogrel (11.375 mg/kg) or vehicle (0.5% CMC-Na) was administered orally to mice for 7 days. Sixty minutes after the last administration, the bleeding time were measured in a mouse tail transection model **(C)**. Data are expressed as means ± SD (*n* = 6). The significance between control and treated groups was calculated by nonparametric Kruskal-Wallis global test using GraphPad Prism. **p < 0.01, ***p < 0.001 compared with the model group.

The effects of naringenin on thrombus formation *in vivo* were also investigated using a FeCl_3_ injury model in rats. Naringenin (200, 400, and 800 mg/kg) was administered orally to rats for 7 days and arterial thrombosis induced by 10% FeCl_3_ on common carotid artery was performed. Thrombus formation was monitored in real time until vessel occlusion using a Transonic Model TS420 flowmeter. As shown in Figure 9A, higher dose of naringenin (800 mg/kg) markedly attenuated 10% FeCl_3_-thrombus formation, as shown by the prolonged time to vessel occlusion, and lower doses of naringenin (200 and 400 mg/kg) produced less inhibition. Importantly, clopidogrel treatment also considerably delayed the vessel occlusion time of rats. Meanwhile, H&E-stained carotid artery showed that complete occlusion of the carotid artery and severe vascular endothelia injury were observed following treatment with 10% FeCl_3_ in vehicle-treated rats. In contrast, naringenin pretreatment was able to diminish thrombus formation and protect against vascular endothelial injury (Figure 9B). These results imply that naringenin possesses antithrombotic activity.

### Effects of Naringenin on Rat Plasma Coagulation

To evaluate the effects of naringenin on rat plasma coagulation, PT, APTT, TT and FIB were measured respectively in rats after oral administration of different doses of naringenin (200, 400, and 800 mg/kg). As illustrated in Table 2, naringenin had no influence on plasma coagulation indexes (*p* > 0.05). In contrast, clopidogrel significantly extended APTT and TT and decreased FIB levels, showing potent anticoagulant effects. Therefore, at the antithrombotic dose used, naringenin did not affect coagulation pathways.

**TABLE 2:** Effects of naringenin on PT, APTT, TT and FIB of rat plasma *in vivo*.

Group	PT (s)	APTT (s)	TT (s)	FIB (g/L)
Control	9.23 ± 0.39	19.80 ± 3.61	50.55 ± 3.95	2.41 ± 0.53
Naringenin 200 mg/kg	9.62 ± 1.24	19.72 ± 2.75	52.70 ± 3.48	2.15 ± 0.50
Naringenin 400 mg/kg	9.67 ± 0.77	19.67 ± 2.98	52.22 ± 2.85	2.20 ± 0.30
Naringenin 800 mg/kg	9.98 ± 0.94	22.88 ± 4.14	54.13 ± 4.99	2.06 ± 0.38
Clopidogrel	10.87 ± 1.68	30.38 ± 5.26***	57.63 ± 4.38**	1.74 ± 0.47*

Data are expressed as means ± SD (*n* = 6). *p < 0.05, **p < 0.01, ***p < 0.001 compared with the control group.

### Effects of Naringenin on Tail Bleeding Time of Mice

To further assess the possible bleeding side effects of naringenin, bleeding time was measured using a tail cutting model in mice after oral administration of different doses of naringenin (280, 560, and 1,120 mg/kg). As illustrated in Figure 9C, naringenin did not significantly prolong the bleeding time at all the concentrations used (*p* > 0.05). However, clopidogrel, which served as the positive control, markedly extended the bleeding time when used at 11.375 mg/kg (*p* < 0.001). These data indicate that naringenin appears to inhibit platelet aggregation and thrombus formation, but not cause a prolonged bleeding time.

## Discussion

Abnormal platelet activation initiates the development of thrombosis, which leads to the progression of ischemic diseases, such as myocardial infarction or stroke. Targeting the inhibition of platelet activation has been shown to be a critical and effective strategy for antithrombotic therapy. However, currently available antiplatelet drugs inevitably increase bleeding risk. Thus, there is a definite need for the development of novel antiplatelet agents with improved safety. It is ideal to prevent atherothrombotic events by long-term intake of nontoxic prophylactic agents, such as food products and nutritional supplements. Several dietary flavonoids have been demonstrated to have beneficial effects in preventing atherothrombotic diseases (Mulvihill et al., 2016). Naringenin is a natural predominant flavonoid abundant in the peel of citrus fruits and has been found to have protective effects against atherothrombotic disorders (Mulvihill et al., 2016). In this study, we demonstrated that naringenin inhibited platelet aggregation both *in vitro* and *ex vivo*. Naringenin also inhibited platelet secretion, integrin αIIbβ3 activation, calcium mobilization and platelet adhesion on collagen-coated surfaces. The inhibitory effects of naringenin were achieved *via* inhibiting PI3K mediated signaling cascades, elevating cGMP levels through suppressing PDE activity, subsequently resulting in VASP phosphorylation at Ser239 by PKA. *In vivo*, naringenin extended arterial occlusion time upon vascular injury without bleeding tendency. Thus, naringenin may represent a safe antiplatelet agent for treatment or prevention of thrombotic disorders.

Platelet agonists activate platelet aggregation *via* various receptors and signaling transduction pathways. Naringenin was found to inhibit ADP-, thrombin- or collagen-induced platelet aggregation with IC_50_ values of around 118, 684 and 236 μM, respectively, indicating that it has increased potency for the inhibition of ADP receptors dependent pathways. ADP exerts its effects through two G-protein-coupled purinoceptors, P2Y_1_ and P2Y_12_ (Beck et al., 2017).The binding of ADP to the Gq-coupled P2Y_1_ receptor activates phospholipase (PLC)-β with consequent mobilization of intracellular calcium, resulting in platelet shape change, granule secretion, and amplification of platelet activation (Gurbel et al., 2015). The binding of ADP to the Gi-coupled P2Y_12_ receptor leads to amplification and stabilization of platelet aggregation. The α subunit of Gi is associated with inhibition of adenylyl cyclase (AC) leading to decrease of cAMP-mediated phosphorylation of VASP. The status of VASP-P regulates glycoprotein IIb/IIIa (GP IIb/IIIa) receptor activation. The βγ subunit induces PI3K, which results in GP IIb/IIIa receptor activation through activation of Akt, ERK and repressor activator protein 1 (Rap1) (Cattaneo, 2015). In the present study, we showed that naringenin inhibited integrin αIIbβ3 activation and PI3K-mediated signaling, and increased cGMP levels and VASP phosphorylation in ADP-induced platelets, indicating that P2Y_12_ receptor as the potential targets of naringenin, although this needs further verification.

Since naringenin largely inhibited ADP-induced platelet activation, further analyses of the signaling proteins involved in the ADP receptors pathway showed that it markedly diminished the activation of PI3K signaling. PI3Kβ, but not PI3Kγ, is recognized to be vial for platelet adhesion to fibrinogen and integrin αIIbβ3-mediated spreading (Canobbio et al., 2009). A previous report indicated that PI3Kp110β was a potential target for antithrombotic drugs because of its critical role in platelet function during arterial thrombosis (Jackson et al., 2005). In our study, naringenin markedly inhibited ADP-induced activation of PI3Kp110β, indicating that PI3K signaling pathway may be involved in the antiplatelet activity of naringenin and that naringenin inhibited platelet adhesion to fibrinogen and integrin αIIbβ3 activation through, at least in part, inhibition of the PI3K pathway. PI3K activation leads to the phosphorylation of the protein Ser/Thr kinase Akt, which is a main essential regulator of platelet granule secretion, secretion-dependent amplification of platelet aggregation and thrombosis (Woulfe et al., 2004; O’Brien et al., 2011). Therefore, targeting PI3K/Akt signaling pathway represents an emerging strategy for the treatment of thrombotic disorder. We found that naringenin decreased the phosphorylation of the PI3K downstream effector Akt at both Thr308 and Ser473 sites in a similar potency on stimulation with ADP. And naringenin had an additive inhibitory effect with the PI3K inhibitor (LY294002) on Akt phosphorylation and platelet aggregation in response to ADP. However, naringenin did not affect the phosphorylation of the Akt substrate GSK3β. These results suggested that the platelet-inhibitory action of naringenin is mediated, at least in part, by inhibiting PI3K/Akt pathway. Moreover, the present data showed that naringenin inhibited the phosphorylaiton of ERK1/2 induced by ADP, indicating that ERK1/2 activity may be associated with the antiplatelet activity of naringenin. Indeed, ERK1/2 plays an important role in platelet activation (Fan et al., 2018). ERK1/2 activation following GPIb stimulation triggers integrin αIIbβ3 activation (Fan et al., 2018). It has been shown that ERK1/2 is activated downstream of P2Y receptors and regulates the generation of Thromboxane A_2_ (TXA_2_) induced by ADP (Garcia et al., 2007). PI3Kβ has been shown to play crucial parts in Gi plus Gz-mediated platelet aggregation and TXA_2_ generation by modulating ERK1/2 phosphorylation (Garcia et al., 2010). In addition, both PI3Kβ and Akt were shown to be an important regulator of ADP-induced ERK1/2 activation (Niba et al., 2013). Therefore, the inhibitory effect of naringenin on ADP-induced ERK1/2 activation could be attributed to naringenin’s negative regulation of P2Y_12_/PI3K/Akt signaling. PI3K and ERK1/2 have been shown to be involved in the influx of extracellular calcium across plasma membrane through the activation of calcium-permeable channel protein and human transient receptor potential channel (hTrp1) coupling with inositol 1, 4, 5-trisphosphate receptor (IP_3_R) type II (IP_3_RII) (Rosado and Sage, 2000; Rosado and Sage, 2001; Mammadova-Bach et al., 2019). Accordingly, the calcium influx across plasma membrane occurred when Fluo 3-loaded washed platelets were stimulated by ADP in the presence of CaCl_2_ (1 mM) due to activating PI3K or ERK1/2 by ADP. Consistent with diminished PI3K and ERK1/2, reduced calcium influx were observed upon treatment with naringenin, indicating that the inhibitory effect of naringenin on ADP-elevated calcium results from the blockade of PI3K signalling.

The activation of integrin αIIbβ3 is the ultimate result of platelet aggregation. Platelet activation by agonist induces dramatic conformational changes of αIIbβ3, which leads to the binding of soluble ligands to αIIbβ3 through a process known as inside-out signaling (Suzuki-Inoue et al., 2001). Binding of multivalent ligands to the activated integrin initiates a series of signaling events known as outside-in signaling that serve to stabilize platelet-platelet interactions and support the process of clot retraction (Huang et al., 2016; Huang et al., 2019). PI3K signaling promotes integrin αIIbβ3 activation through activating Akt, ERK and Rap1 (Gurbel et al., 2015). In our study, naringenin inhibited **i**ntegrin αIIbβ3-mediated inside-out signaling, as evidenced by suppressing fibrinogen binding of single platelets. The ability of naringenin to inhibit **i**ntegrin αIIbβ3-mediated inside-out signaling was consistent with its ability to inhibit PI3K signaling. Similarly, naringenin was found to inhibit platelet spreading on fibrinogen and clot retraction, which are dependent on **i**ntegrin outside-in signaling. These results indicated that naringenin involves acting on not only platelet inside-out signaling but also outside-in signaling, both of which are known to be largely modulated by PI3K (Canobbio et al., 2009; Gurbel et al., 2015).

In our study, naringenin was found to increase platelet cGMP levels and VASP phosphorylation at Ser239 which is preferentially phosphorylated by PKG. Whereas, naringenin did not show any effects on the elevation of cAMP levels or the phosphorylation of VASP at Ser157 which is preferred by PKA. The important role of PKG for the phosphorylation of VASP at Ser239 is well established, but some debate recently appears as to whether this site (Ser239) may also be phosphorylated by PKA-dependent signaling (Walter and Gambaryan, 2004). cGMP has been shown to activate cAMP-PKA signaling pathway by inhibiting phosphodiesterase 3 (Jang et al., 2002). PKG-deficient mice or patients also showed PKA-dependent cellular responses to cGMP (Eigenthaler et al., 1993; Sausbier et al., 2000). Furthermore, H89, a potent PKA inhibitor with a Ki for PKA 10 times less than its Ki for PKG (Chijiwa et al., 1990), was shown to inhibit cGMP-induced VASP phosphorylation (Burkhardt et al., 2000), suggesting that PKA is possible involved in cGMP-induced VASP phosphorylation. Our data demonstrated that naringenin-mediated VASP phosphorylation at Ser239 was reversed by a PKA inhibitor (H89) treatment, indicating that the phosphorylation of this site in VASP by naringenin is predominantly mediated through PKA instead of PKG. In addition, naringenin induced-inhibition of platelet aggregation was also reversed by the PKA inhibitor but not by the PKG inhibitor, indicating that naringenin-induced PKA activation is required for naringenin-induced platelet inhibition. Our data are consistent with a previous report showing that PKA plays a predominant role in the cGMP-induced phosphorylation of VASP and platelet inhibition in human platelets (Li et al., 2003b). In the previous report, the phosphorylation of VASP at Ser239 induced by cGMP analogs or NO donors was inhibited by several different specific PKA inhibitors (Li et al., 2003a). Although we conclude a predominant role of PKA but not PKG in naringenin-induced VASP at Ser239 in rat platelets, we do not exclude the possible involvement of PKG in VASP phosphorylation following an increase in intracellular cGMP. It is possible to fail to detect the contribution of PKG to Ser239 under our experiment conditions, as the predominant role of PKA could mask the inhibitory effects of PKG inhibitors.

*In vivo* antithrombotic role of naringenin was further demonstrated in the FeCl_3_-induced rat carotid arterial thrombus model, in which it prevented thrombus formation and vessel occlusion. Generally, existing antithrombotic/platelet drugs, such as clopidogrel and aspirin, are associated with bleeding complications. Interestingly, naringenin, at the equivalent doses employed in FeCl_3_-induced rat thrombosis model, did not significantly prolong the bleeding time by a tail cutting model in mice. In contrast, clopidogrel caused much more bleeding. Previous studies have shown that compound targeting PI3K do not cause prolonged bleeding time and PI3Kβ inhibitors in combination with anticoagulants induce much less bleeding than P2Y_12_ antagonists or aspirin (Jackson et al., 2005; Jackson and Schoenwaelder, 2012). Similarly naringenin, an inhibitor partially targeting PI3K/Akt pathway in platelets, exhibited potent antithrombotic efficacy and weak bleeding side effect.

In conclusion, naringenin inhibits platelet activation *via* the suppression of PI3K mediated signaling cascades and PDE activity, followed by increasing cGMP levels and PKA-mediated VASP phosphorylation on ADP-induced stimulation. This bioflavonoid exhibits potent antithrombotic efficacy while without significant bleeding diathesis. Therefore, our data indicate that naringenin may be developed as a potent and safe therapeutic agent against abnormal platelet activation-related thrombotic diseases.

## Data Availability

The original contributions presented in the study are included in the article/Supplementary Material, further inquiries can be directed to the corresponding authors.
