# Supplementary figures and images for: Naringenin Inhibits Platelet Activation and Arterial Thrombosis Through Inhibition of Phosphoinositide 3-Kinase and Cyclic Nucleotide Signaling
Source: Front Pharmacol. 2021 Aug 12;12:722257. doi: 10.3389/fphar.2021.722257 (PMC8406801; doi:10.3389/fphar.2021.722257)

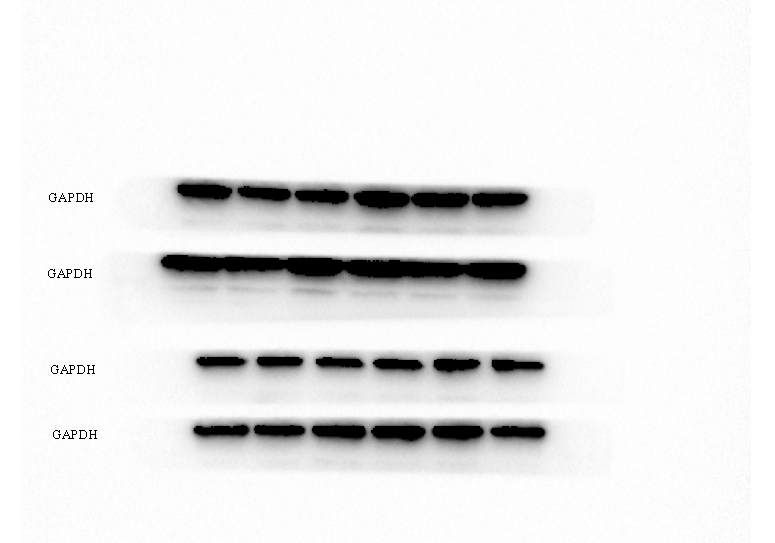

Supplement: Supplementary file 1 [file DataSheet2.ZIP › uncropped image of western blots/Ex vivo/GAPDH.tif]

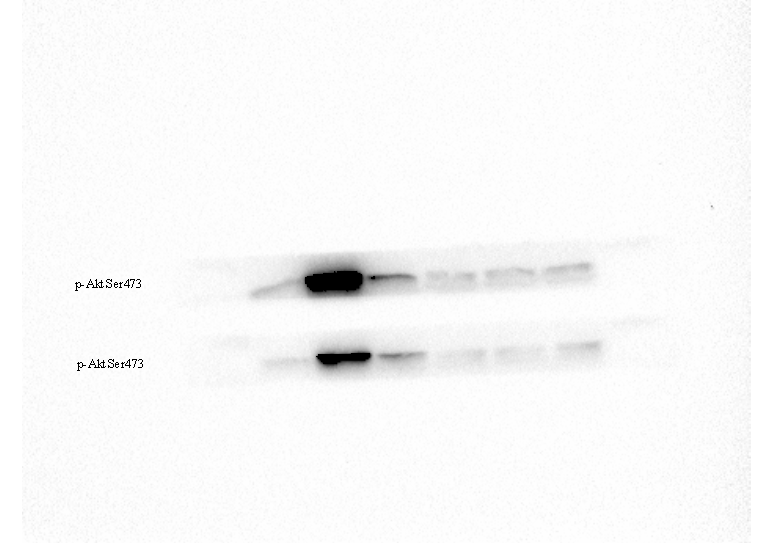

Supplement: Supplementary file 1 [file DataSheet2.ZIP › uncropped image of western blots/Ex vivo/p-Akt Ser473.tif]

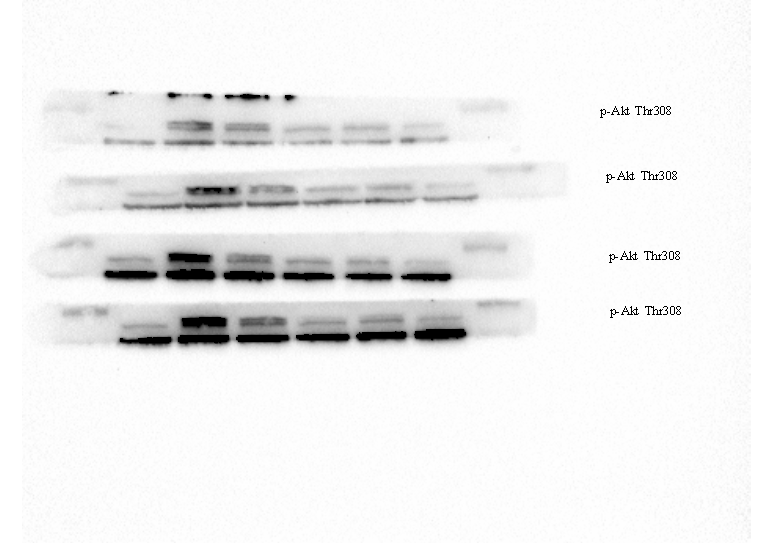

Supplement: Supplementary file 1 [file DataSheet2.ZIP › uncropped image of western blots/Ex vivo/p-Akt Thr308.tif]

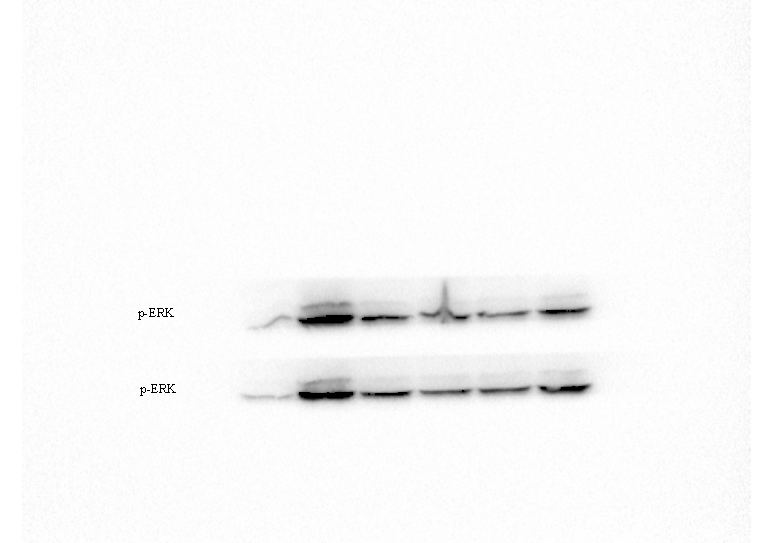

Supplement: Supplementary file 1 [file DataSheet2.ZIP › uncropped image of western blots/Ex vivo/p-ERK.tif]

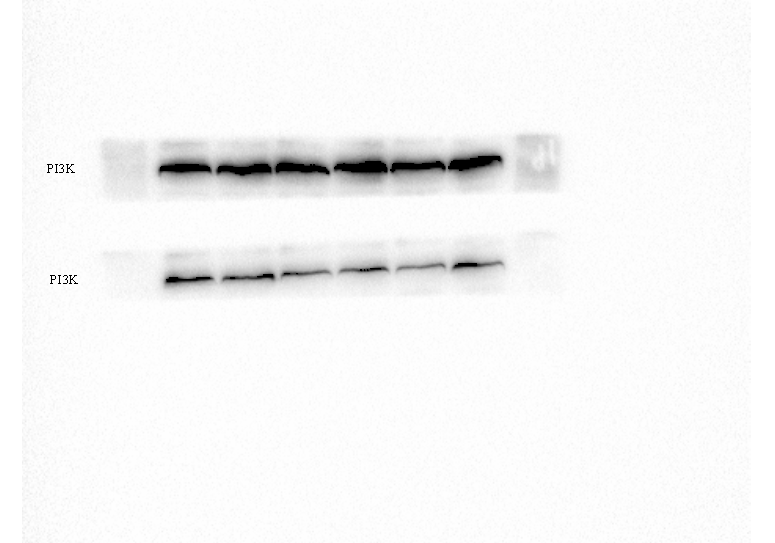

Supplement: Supplementary file 1 [file DataSheet2.ZIP › uncropped image of western blots/Ex vivo/PI3K.tif]

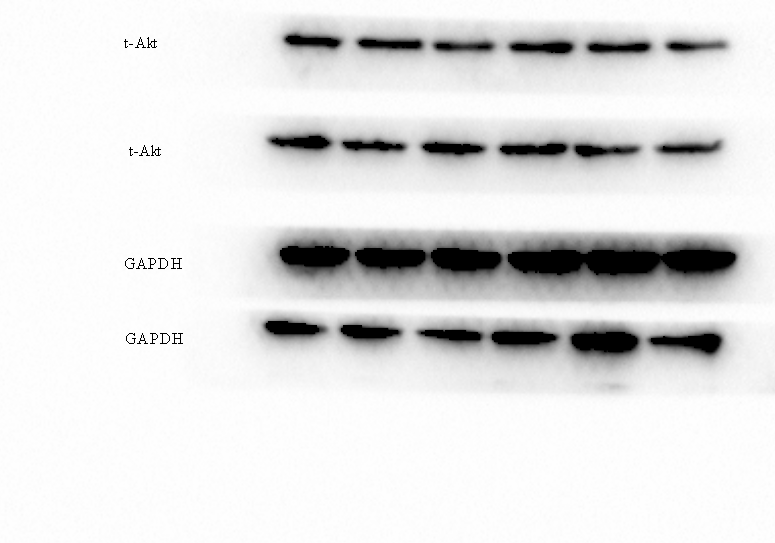

Supplement: Supplementary file 1 [file DataSheet2.ZIP › uncropped image of western blots/Ex vivo/t-Akt.tif]

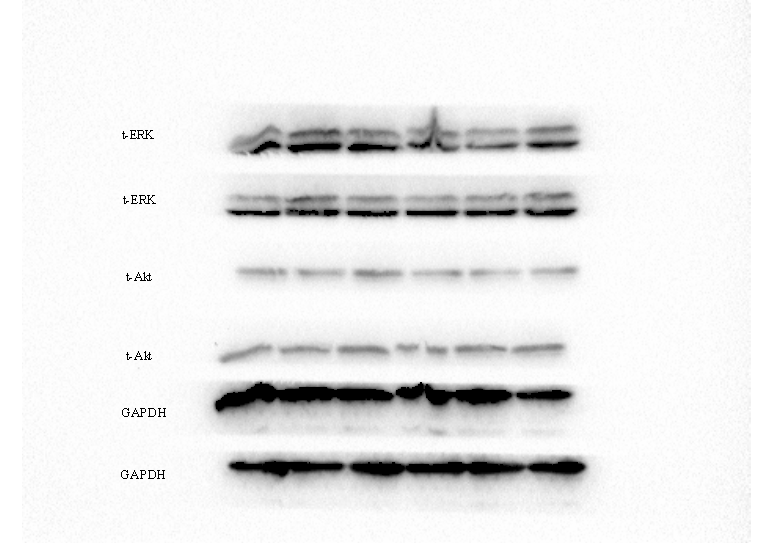

Supplement: Supplementary file 1 [file DataSheet2.ZIP › uncropped image of western blots/Ex vivo/t-ERK.tif]

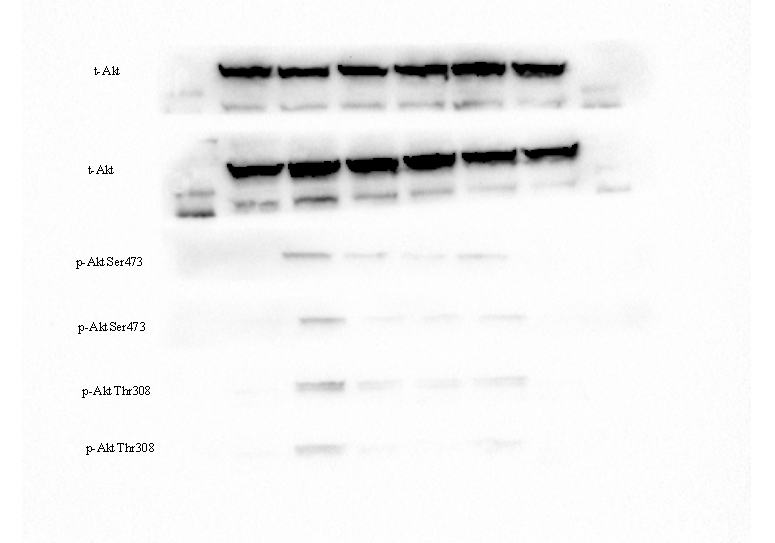

Supplement: Supplementary file 1 [file DataSheet2.ZIP › uncropped image of western blots/In vitro/Akt.tif]

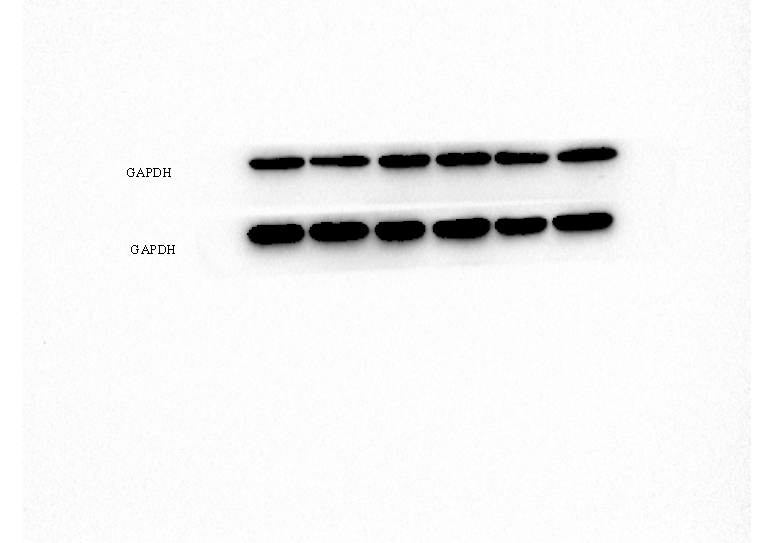

Supplement: Supplementary file 1 [file DataSheet2.ZIP › uncropped image of western blots/In vitro/GAPDH 6.tif]

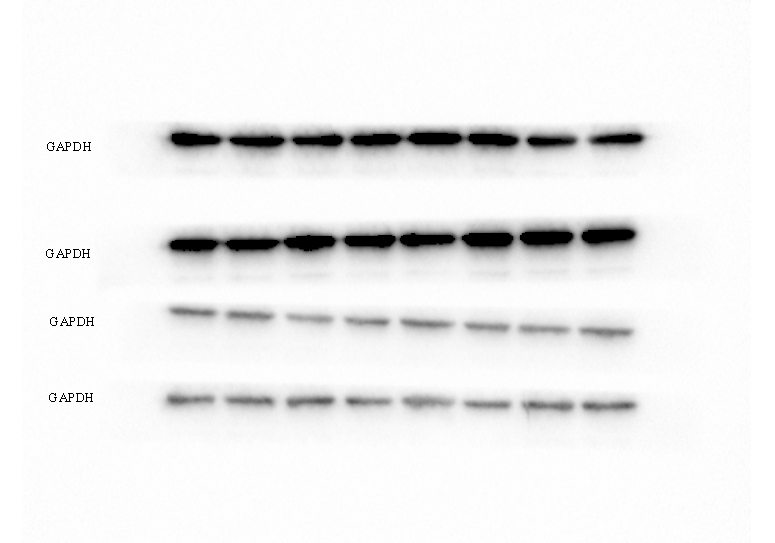

Supplement: Supplementary file 1 [file DataSheet2.ZIP › uncropped image of western blots/In vitro/GAPDH 8.tif]

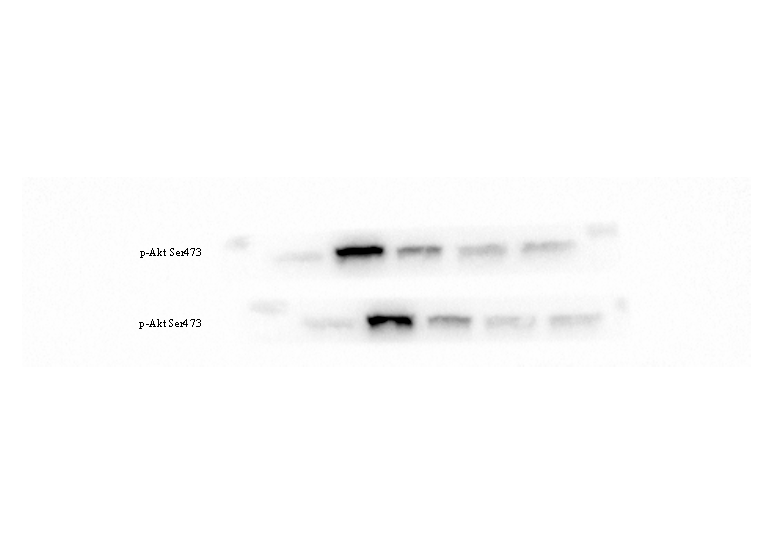

Supplement: Supplementary file 1 [file DataSheet2.ZIP › uncropped image of western blots/In vitro/p-Akt Ser473 5.tif]

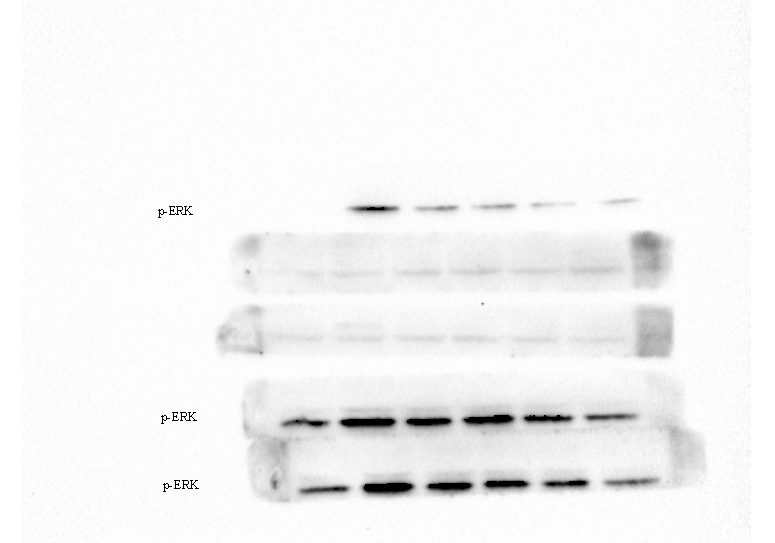

Supplement: Supplementary file 1 [file DataSheet2.ZIP › uncropped image of western blots/In vitro/p-ERK 1.tif]

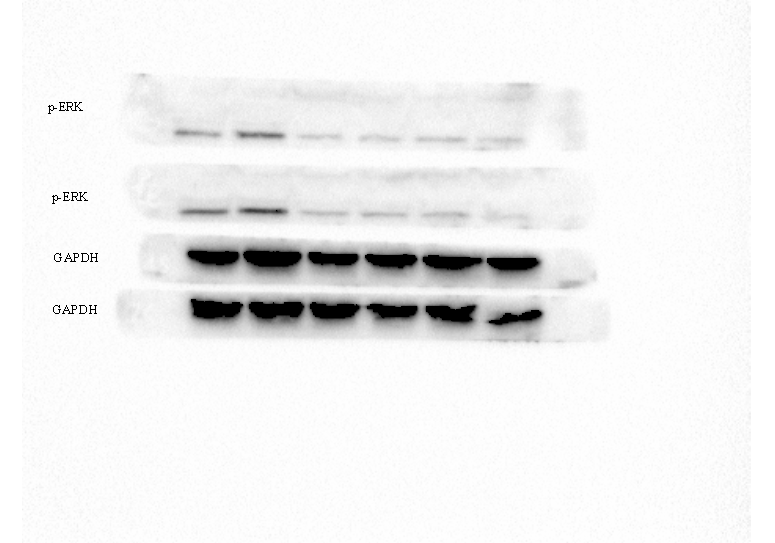

Supplement: Supplementary file 1 [file DataSheet2.ZIP › uncropped image of western blots/In vitro/p-ERK 2.tif]

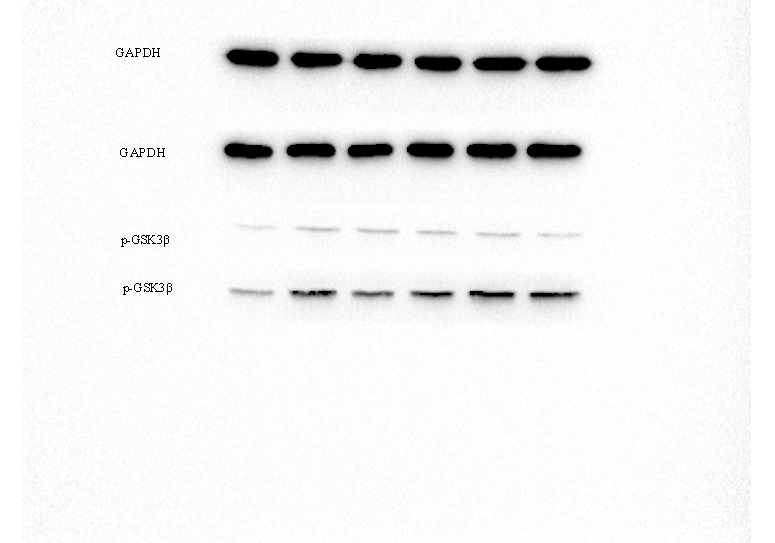

Supplement: Supplementary file 1 [file DataSheet2.ZIP › uncropped image of western blots/In vitro/p-GSK3B.tif]

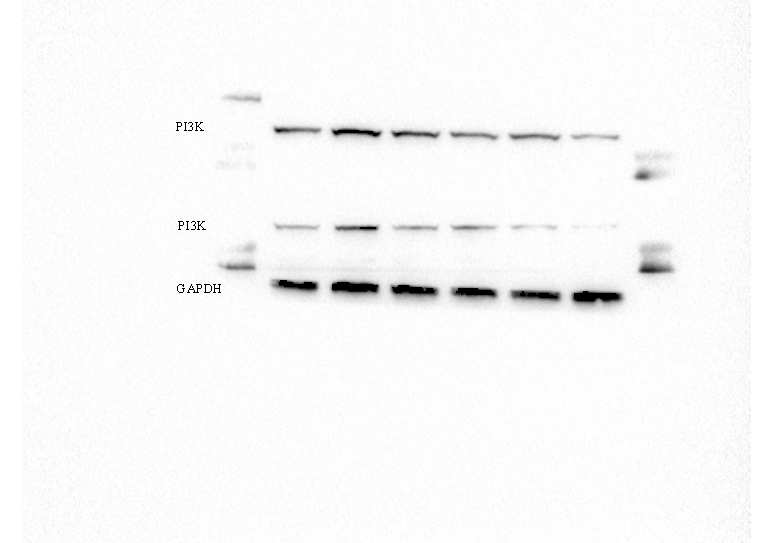

Supplement: Supplementary file 1 [file DataSheet2.ZIP › uncropped image of western blots/In vitro/PI3K.tif]

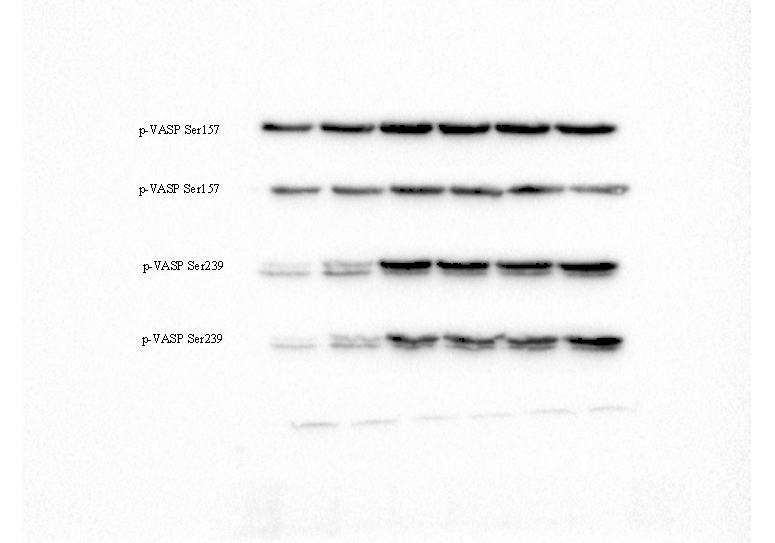

Supplement: Supplementary file 1 [file DataSheet2.ZIP › uncropped image of western blots/In vitro/p-VASP 1.tif]

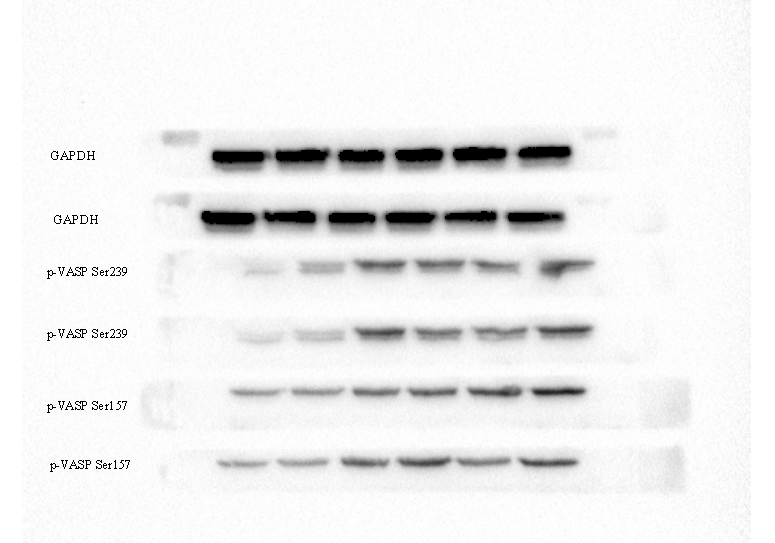

Supplement: Supplementary file 1 [file DataSheet2.ZIP › uncropped image of western blots/In vitro/p-VASP 2.tif]

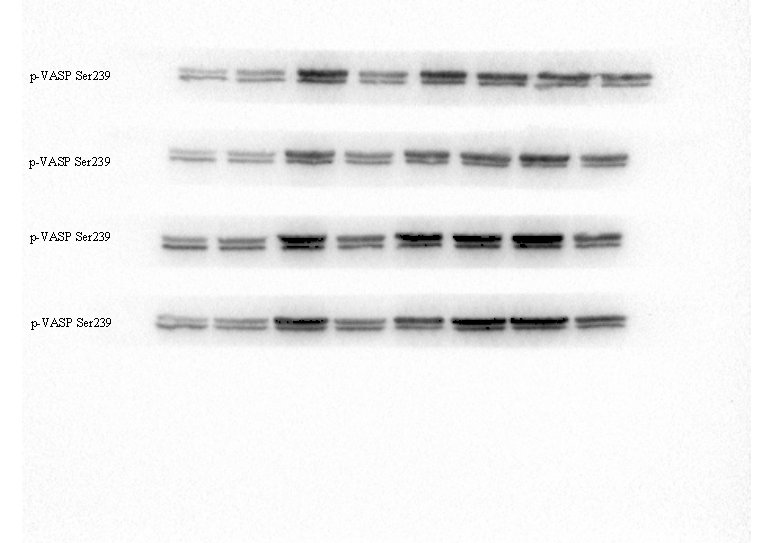

Supplement: Supplementary file 1 [file DataSheet2.ZIP › uncropped image of western blots/In vitro/p-VASP Ser239.tif]

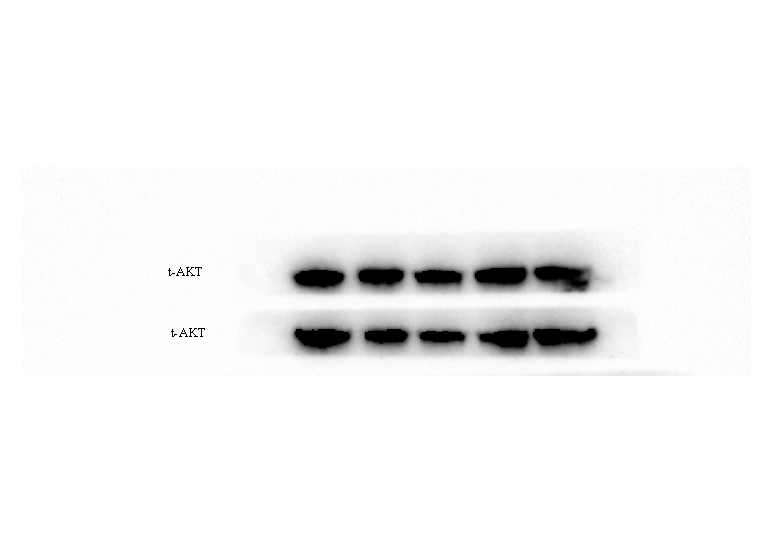

Supplement: Supplementary file 1 [file DataSheet2.ZIP › uncropped image of western blots/In vitro/t-Akt 5.tif]

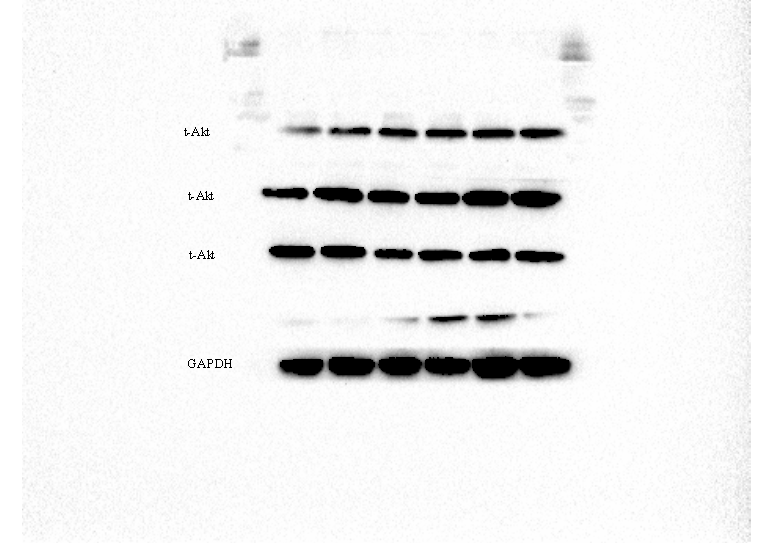

Supplement: Supplementary file 1 [file DataSheet2.ZIP › uncropped image of western blots/In vitro/t-Akt 6.tif]

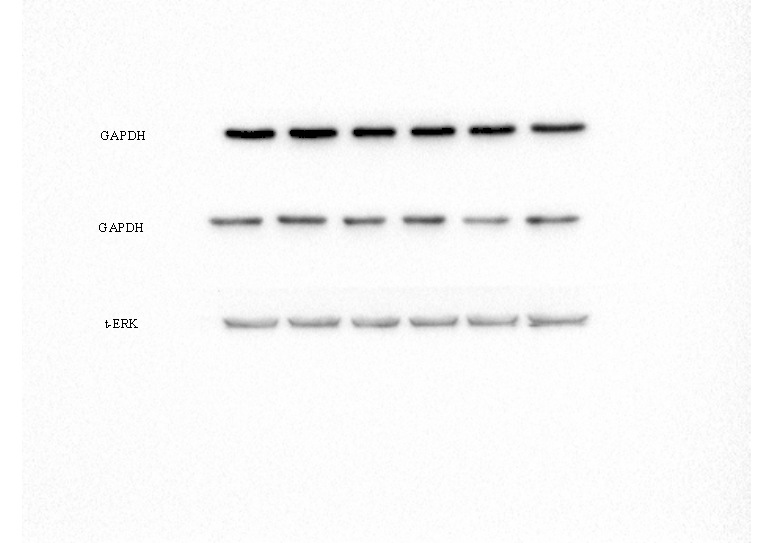

Supplement: Supplementary file 1 [file DataSheet2.ZIP › uncropped image of western blots/In vitro/t-ERK.tif]

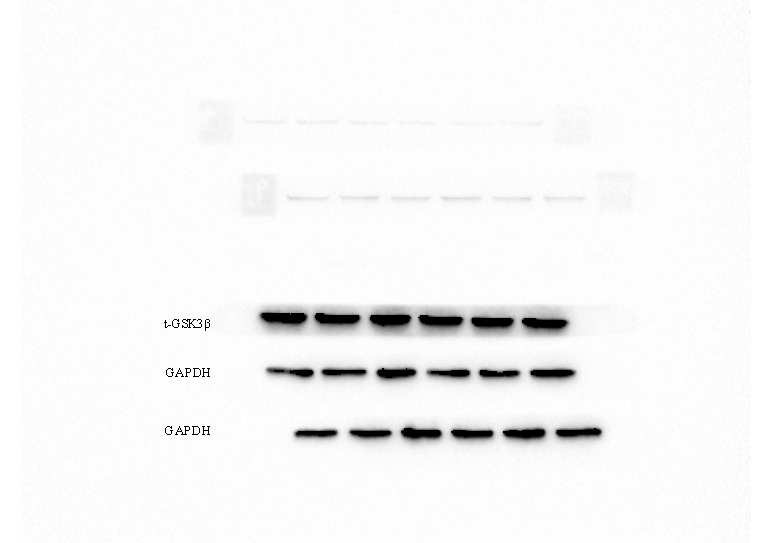

Supplement: Supplementary file 1 [file DataSheet2.ZIP › uncropped image of western blots/In vitro/t-GSK3B.tif]
