# Supplementary material for: Naringenin Inhibits Platelet Activation and Arterial Thrombosis Through Inhibition of Phosphoinositide 3-Kinase and Cyclic Nucleotide Signaling
Source: Front Pharmacol. 2021 Aug 12;12:722257. doi: 10.3389/fphar.2021.722257 (PMC8406801; doi:10.3389/fphar.2021.722257)
Supplement: Supplementary file 3 [file Image1.pdf]

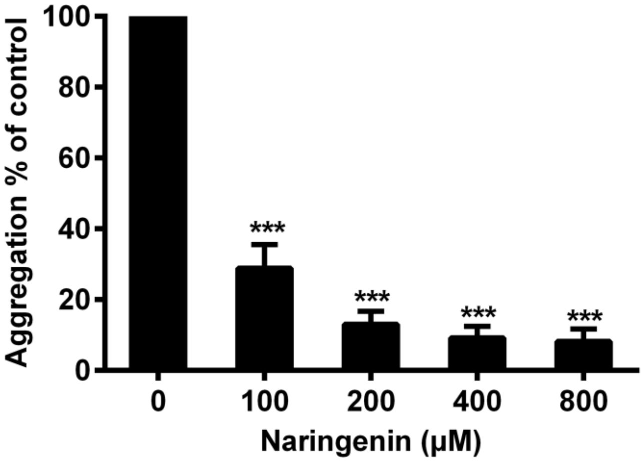

FIGURE S1 Naringenin inhibits ADP-induced platelet aggregation *in vitro*. Rat washed platelet aggregation performed with vehicle or various concentrations of naringenin was recorded after stimulation with 5 μmol/L ADP. Data are expressed as means ± SD (*n*=6), the level of aggregation obtained at 5 min with vehicle-treated samples (0 μmol/L) was taken as 100%. ***P<0.001 compared with the vehicle control group.
