# Supplementary material for: Naringenin Inhibits Platelet Activation and Arterial Thrombosis Through Inhibition of Phosphoinositide 3-Kinase and Cyclic Nucleotide Signaling
Source: Front Pharmacol. 2021 Aug 12;12:722257. doi: 10.3389/fphar.2021.722257 (PMC8406801; doi:10.3389/fphar.2021.722257)
Supplement: Supplementary file 1 [file DataSheet2.ZIP › uncropped image of western blots/Ex vivo/illustration.doc]

1	2	3	4	5	6

1: ADP 5μM (-)

2: ADP 5μM (+)

3: ADP 5μM (+), Naringenin 200mg/kg (+)

4: ADP 5μM (+), Naringenin 400mg/kg (+)

5: ADP 5μM (+), Naringenin 800mg/kg (+)

6: ADP 5μM (+), Clopidogrel (+)
