# Supplementary material for: Naringenin Inhibits Platelet Activation and Arterial Thrombosis Through Inhibition of Phosphoinositide 3-Kinase and Cyclic Nucleotide Signaling
Source: Front Pharmacol. 2021 Aug 12;12:722257. doi: 10.3389/fphar.2021.722257 (PMC8406801; doi:10.3389/fphar.2021.722257)
Supplement: Supplementary file 1 [file DataSheet2.ZIP › uncropped image of western blots/In vitro/illustration.doc]

1	2	3	4	5	6

1: ADP 10μM (-), Naringenin (-)

2: ADP 10μM (+), Naringenin (-)

3: ADP 10μM (+), Naringenin 100μM (+)

4: ADP 10μM (+), Naringenin 200μM (+)

5: ADP 10μM (+), Naringenin 400μM (+)

6: ADP 10μM (+), Naringenin 800μM (+)

1	2	3	4	5

1: ADP 10μM (-), Naringenin 200μM (-), LY294002 5μM (-)

2: ADP 10μM (+),Naringenin 200μM (-), LY294002 5μM (-)

3: ADP 10μM (+),Naringenin 200μM (+), LY294002 5μM (-)

4: ADP 10μM (+),Naringenin 200μM (-), LY294002 5μM (+)

5: ADP 10μM (+),Naringenin 200μM (+), LY294002 5μM (+)

1	2	3	4	5	6	7	8

1: ADP 10μM (-)

2: ADP 10μM (+)

3: ADP 10μM (+), Naringenin 800μM (+), PKG inhitor (+)

4: ADP 10μM (+),Naringenin 800μM (+), PKA inhitor (+)

5: ADP 10μM (+),Naringenin 800μM (+), PKC inhitor (+)

6: ADP 10μM (+), Naringenin 800μM (+)

7: ADP 10μM (+), Naringenin 400μM (+)

8: ADP 10μM (+), Naringenin 200μM (+)
